# Effect of curing temperature freeze–thaw failure mechanism and damage model of equal-strength air-entrained concrete

**DOI:** 10.1371/journal.pone.0312890

**Published:** 2024-12-26

**Authors:** YongHe Liu, Bo Yang, Aojun Guo

**Affiliations:** 1 Civil Engineering Department, Lanzhou Jiaotong University, Lanzhou, China; 2 National and Provincial Joint Engineering Laboratory of Road & Bridge Disaster Prevention and Control, Lanzhou Jiaotong University, Lanzhou, China; Mirpur University of Science and Technology, PAKISTAN

## Abstract

The Belt and Road strategy has significantly advanced the scale of infrastructure construction in the Qinghai–Tibet Plateau permafrost area. Consequently, this demands higher requirements on the strength and frost resistance of concrete (FRC) cured under low-temperature and negative-temperature conditions. Accordingly, in this study, tests on the mechanical properties and FRC were conducted under standard curing, 5 °C curing, and −3 °C curing conditions. The pore structure characteristics of concrete subjected to freeze–thaw (F–T) damage (FTD) under different curing methods were analyzed using nuclear magnetic resonance. The study results show that when the air content is constant, the compressive strength of concrete (CSC) tends to decrease with the curing temperature. Moreover, the occurrence of an age lag phenomenon is evident. The compressive strength of concrete cured under standard curing for 28-d was comparable to that achieved by concrete cured at 5 °C curing for 56-d and at −3 °C curing for 84-d. Under the same curing conditions, the CSC decreases with increasing air content. Observations revealed that with the air content in the concrete set at 0.08%, the material’s compressive strength was at its minimum. As the number of F–T cycles increases, the concrete transverse relaxation time (T_2_) curve shifts to the right, and the proportion of both harmful and multi-harmful pores increases. Based on the same CSC under different curing methods, the FRC under 5 °C curing and −3 °C curing conditions is considerably lower than that under standard curing conditions. Moreover, the FRC exhibits an increasing and then a decreasing trend with increasing air content. Concrete exhibits the best frost resistance when the air content is 3.6%. It was established that an optimal range exists for air content in concrete. If the air content is too low, there is only a slight improvement in the FRC. Conversely, if the air content was excessively high, it leads to a significant decrease in frost resistance. Further, this study establishes an FTD model for concrete under 5 °C curing and −3 °C curing conditions considering the compressive strength factors of concrete under standard curing conditions for 28-d. This study is anticipated to be used as reference for determining the FRC cured under different temperatures.

## 1. Introduction

With rapid economic development, the scale of infrastructure construction in the world is increasing every year. The Qinghai-Tibet Plateau permafrost region [1] was home to a variety of concrete structures. These include railways, highways, industrial and civil buildings, and oil and gas pipelines. The annual average temperature in this region is ≤ −3 °C. Permafrost is different from cold soil (soil with negative temperature but with no ice formed), seasonally frozen soil (frozen in winter only), and biennial frozen soil (frozen in winter and remains frozen for one or two years). In constructing concrete structures in this region, maintaining the molds at low temperatures (less than 5 °C) is necessary to avoid disturbing the permafrost [2]. Under negative-temperature conditions, the hydration reaction of concrete is insufficient, resulting in inadequate compressive strength in the early curing stages. This further leads to a significant difference between the strength of concrete cured under the foregoing condition and that of concrete under standard curing conditions. If construction is conducted according to the age of concrete under standard curing conditions, hidden dangers may threaten project quality and safety [3–5].

Permafrost is affected by concrete construction, heat, and heat released from hydration, spatial variability and thermal disturbance change the physical field and upper limit of frozen soil in permafrost regions, affecting the thermal stability of deep level permafrost. This accelerates the degradation of frozen soil, possibly transforming permafrost into seasonally frozen soil [6–8]. Therefore, the main cause of damage to concrete structures in terms of durability is a lack of frost resistance [9–11].

Many scholars have conducted experiments and research on the factors affecting the frost resistance of concrete (FRC). Beskopylny et al. [12] investigated how various manufacturing processes affect concrete’s freezing resistance. Their findings revealed that concrete made using vibration centrifugal methods demonstrates superior freezing resistance and increased durability. Rashidi et al. [13] investigated the impact of volcanic ash, particularly kaolin nanotubes, on cement mortar’s properties over the long term. These properties encompass physical and mechanical traits, permeability, durability, and microstructure, all critically tested under repeated freeze-thaw cycles. The research findings indicate that the inclusion of kaolin nanotubes significantly improves the frost resistance of cement mortar, surpassing that of standard mortar. Tanyildizi et al. [14] examined the freezing resistance of concrete exposed to sulfuric acid and salt. The research indicated that concrete with 500 kg/m^3^ of cement demonstrated superior resilience under freeze-thaw cycles and 1% acid attack. Notably, this resilience manifested as maximum compressive strength, rapid ultrasonic pulse speed, and negligible weight loss. Raczkiewicz et al. [15] examined three reinforced concrete specimens: standard concrete, and two with different steel fiber contents (0.25% and 0.5% of concrete volume). They aimed to identify key factors that cause reinforced concrete to degrade under salt invasion and freeze-thaw cycles. Their research showed that adding steel micro-reinforced fibers significantly improves the material’s corrosion resistance. In their research, Samuel et al. [16] investigated the correlation between cement composition and concrete’s frost resistance. Their findings indicated that concrete made with composite cement demonstrated reduced resistance to FTCs. Adding supplementary cementitious materials like fly ash [17] and granulated blast furnace slag [18, 19] to concrete can replace a portion of the cement. This leads to an increased production of hydrated calcium silicate gel, which fills the pores, thus making the concrete denser. Consequently, this enhances the concrete’s frost resistance and durability. Abdulaziz et al. [20] assessed the freeze-thaw resilience of steel fiber-reinforced rubber concrete, particularly for use in flexible concrete pavements. Their research revealed that this concrete variant withstood 56 FTCs without suffering internal damage or loss of mechanical properties. These results reinforce the suitability of steel fiber-reinforced rubber concrete in harsh freeze-thaw environments, positioning it as a sustainable and durable alternative to traditional asphalt in pavement construction.

Many scholars have proposed FTD models (FTDMs) based on the study of factors affecting the FRC. Berto L and his team [21] studied the effects of freeze-thaw cycles on the bonding properties of standard concrete. To tackle this issue, they created a new bond stress-slip model. When this model’s predictions were matched against actual test results, it accurately predicted the decrease in bond strength. This model proves especially useful in the structural analysis of reinforced concrete affected by freeze-thaw cycles. Bai et al. [22] introduced the fatigue damage theory according to the basic principles of irreversible thermodynamics and continuum damage mechanics. Subsequently, they established macro-FTD and micro-FTD equations for aeolian sand concrete. Pei et al. [23] proposed an F–T deterioration model for concrete based on the Chaboche fatigue damage theory. Manawadu et al. [24] analyzed the freeze-thaw resilience of shotcrete’s interface bond under tension. They crafted a probabilistic damage model based on a three-parameter Weibull distribution, linking the number of freeze-thaw cycles to damage parameters. This model proficiently assesses the durability against freeze-thaw effects and forecasts the service life of the interface bond in shotcrete. Zaghian et al. [25] utilized the finite element software DIANA to analyze the structural responses of reinforced concrete piers against freeze-thaw cycles, corrosion, and service loads throughout their lifespan. To gauge the proposed methods’ effectiveness for various damage mechanisms, they compared them with experimental data sourced from the literature.

Previous studies have found that the addition of an appropriate dosage of air entrainment agent can cause the internal structure of concrete to produce numerous minute bubbles, improving the FRC[26–28]. In addition to mechanical strength, some researchers believe that the FRC mainly depends on the bubble spacing coefficient and air content [29]. Hasholt et al. [30] conducted frost resistance tests on different types of concrete in which the FRC depended on the total surface area of the pores inside the concrete. Blikharskyy et al. [31] analyzed the effects of air entraining agents on mortar mixtures, particularly their influence on the compressive strength of hardened mortar. The study revealed that higher levels of air entrainment agents decrease both density and compressive strength in mortar, yet simultaneously improve its resistance to freeze-thaw cycles. In their research, Kia A. [32] determined that concrete featuring anti-blocking pervious pavement demonstrates significant resilience against freeze-thaw degradation, and this is achieved without the need for air entrainment agents. Anwar et al. [33] examined how air entraining agents (AEAs) and different sample surface areas (top, bottom, and sides) affect chloride ion penetration and diffusion in concrete. Their findings indicated a reverse relationship between the use of AEAs, as well as the water-cement ratio, and concrete performance. Additionally, changes in the water-cement ratio, cement content, and AEA amounts markedly affect the diffusion coefficient. Konstantinos Stergiou [34] outlined how artificial intelligence algorithms, such as neural networks, random forests, and support vector machines, are employed to predict FRC and service life.

The foregoing shows that most studies focus on the theory of FRC under standard curing conditions. However, the research on the FRC cured under low-temperature and negative-temperature conditions is limited. And the change of compressive strength and porosity of concrete under low negative temperature curing condition remains to be studied. In formulating the FTDM, most studies were based on the relative dynamic elastic modulus (RDEM) of concrete; the number of FTCs was used as an independent variable. Few FTDMs consider the compressive strength of concrete (CSC).

Accordingly, in this study, the mechanical properties and F–T resistance of concrete under standard curing, 5 °C curing, and −3 °C curing conditions are tested. The effects of curing temperature and air content on the CSC and FRC are explored. The pore structure characteristics of concrete cured under different conditions and damaged by F–T are analyzed by nuclear magnetic resonance (NMR). In addition, based on the FTDM of concrete proposed in previous studies, an FTDM of concrete cured under different curing conditions considering the 28-d CSC under standard curing conditions is established. This study is anticipated to provide a theoretical basis for maintaining and constructing concrete under 5 °C curing and −3 °C curing conditions. The F–T fatigue damage to concrete cured under the foregoing conditions is also predicted. Research on the FTD of concrete, particularly when it maintains consistent strength in low and sub-zero temperatures, was crucial. This was highly pertinent to the construction and maintenance of bored pile foundations in the Qinghai-Tibet region. And according to the change of concrete compressive strength, frost resistance and porosity under low negative temperature curing environment, it can provide theoretical guidance for the construction of concrete structure under low negative temperature curing environment (Table 1 is a collection of all abbreviations for this article).

**Table 1:** Noun abbreviations.

FRC	frost resistance of concrete	DEM	dynamic elastic modulus
CSC	compressive strength of concrete	NMR	nuclear magnetic resonance
FTD	freeze–thaw damage	AEA	air-entraining agent
F-T	freeze–thaw	RDEM	relative dynamic elastic modulus
FTDM	freeze–thaw damage model	FTCs	freeze–thaw cycles
MLR	mass loss rate		

## 2. Materials and methods

### 2.1. Test of ingredients and mixture proportions ratio

#### 2.1.1. Concrete raw materials

**Fig 1:**
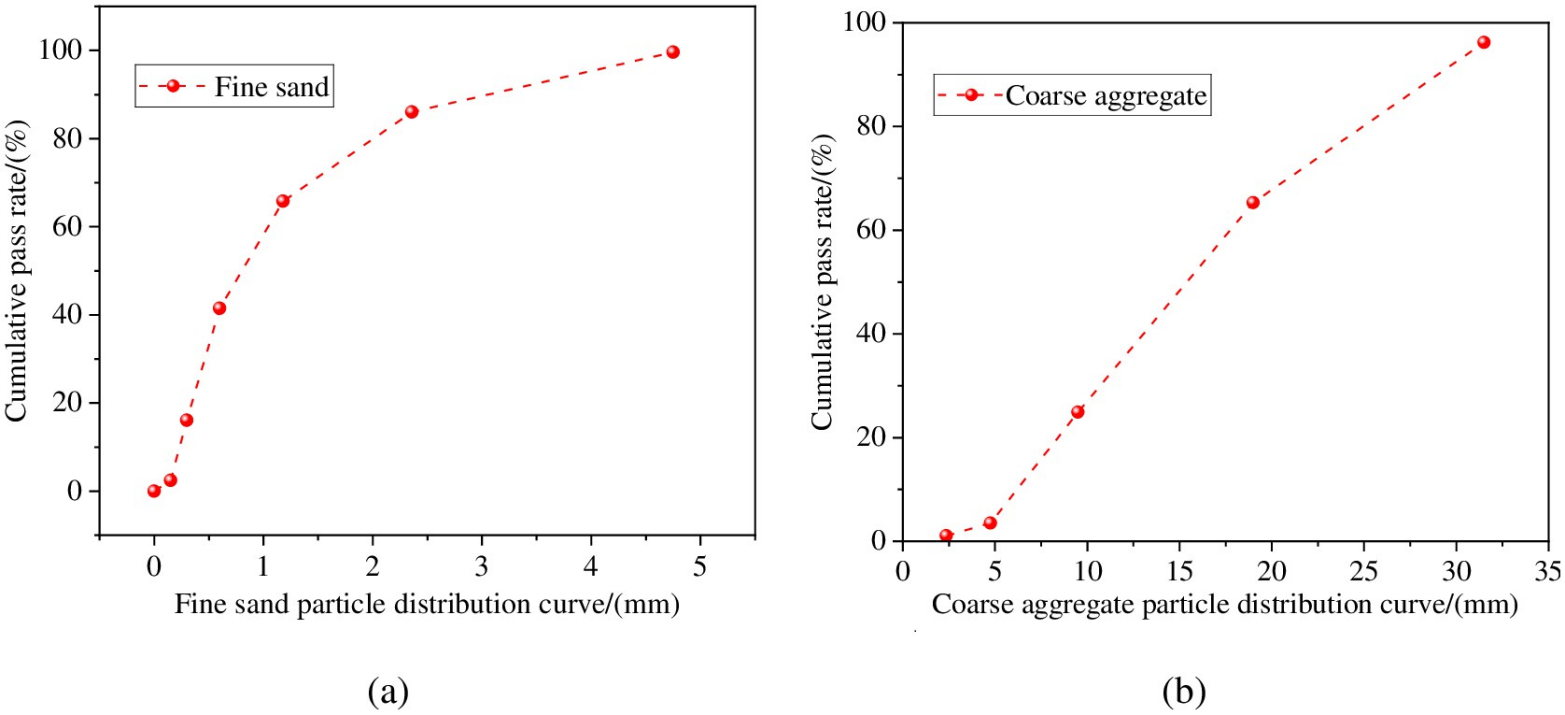
Particle distribution curve: (a) Fine sand; (b) Coarse sand.

The cement (C) used is Gansu Qilian Mountain P.O 42.5-grade ordinary silicate cement; The cement density is 2.9 g/cm^3^, Technical specifications are outlined in Table 2. The ordinary Portland cement utilized in the test adhered to the General Purpose Portland Cement standards [35] (GB 175–2007), ensuring compliance with all necessary criteria. The continuous gradation, crushing index, and apparent density of crushed stone (G) particles were 5–31.5 mm, 7.04%, and 2790 kg/m^3^, respectively. According to the Pebbles and gravel for Construction (GB/T 14685–2022) standard [36], the rubble meets the requirements. Fine sand (S) was used; its fineness modulus, apparent density, bulk density, and mud content were 2.15, 2620 kg/m^3^, 1515 kg/m^3^, and 1.47%, respectively. The fineness modulus of fine sand is 2.8, which belongs to medium sand and is well graded. According to the Construction sand (GB/T14684-2022) standard [37], the sand meets the requirements. Fig 1 shows the particle distribution curves of fine sand and coarse aggregate. Mineral powder (MP, grade II) and fly ash (FA, grade I) were also used. The performance indexes of mineral powder are shown in Table 3. The properties of fly ash are shown in Table 4. The MP admixture comprised a water-reducing agent (WR) and an air-entraining agent (AEA). The AEA content is the percentage of gelled material. High-performance polycarboxylic acid was also used as a WR. The superplasticizer is a light yellow viscous liquid with a water reduction rate of 25%. The water (W) utilized for mixing was tap water, the PH value is 6.2.

**Table 2:** Technical specifications of cement.

Cement	Specific surface area (m^2^/kg)	Setting time (min)		Flexural strength (MPa)		Compressive strength (MPa)	
		Initial	Final	3-d	28-d	3-d	28-d
P.O 42.5	360	95	140	5.5	8.9	23.4	46.6

**Table 3:** Performance index of mineral powder.

Index	Firing loss(%)	Moisture content(%)	Specific surface area(m^2^/kg)	SO_3_ content(%)	Cl^-^ content(%)
National standard	≤3.0	≤1.0	≥400	≤3.2	≤0.06
Measured value	2.0	0	450	0.28	0.015

**Table 4:** Performance index of fly ash.

Index	fineness	Water demand ratio	Firing loss	Water content	SO_3_ content
National standard/(%)	≤12	≤95	≤5	≤1	≤3
Measured value/(%)	5.7	90.6	3.0	0	0.46

#### 2.1.2. Concrete mix ratio

For this test, a concrete mix featuring a water-binder ratio of 0.38 was chosen. This selection adhered to the actual construction project requirements and standard design specifications for ordinary concrete mixes [38], as outlined in Table 5. The A-1 series is the reference concrete, that is, concrete without an AEA. The content of air-entraining agents in the A-2, A-3, and A-4 samples was established at 0.03%, 0.06%, and 0.08% of the gelling material, respectively. The A-2, A-3, and A-4 series had AEA contents of 0.03%, 0.06%, and 0.08%, respectively. In actual mixing, to maintain the concrete’s workability, the slump range was kept between 180–220 mm, while the expansion was consistently regulated to stay within 500–550 mm. And the fluidity is good. There is a certain cohesive force between the composition of the new concrete, no stratification and segregation phenomenon, and the water retention is also good.

**Table 5:** Concrete mix proportions.

w/b	Group	W (kg/m^3^)	C (kg/m^3^)	MP (kg/m^3^)	FA (kg/m^3^)	S (%)	WR (%)	AEA (%)	Air content (%)
0.38	A-1	150	276	59	59	46	1.5	0	1.5
	A-2	150	276	59	59	46	1.5	0.03	3.6
	A-3	150	276	59	59	46	1.5	0.06	6.4
	A-4	150	276	59	59	46	1.5	0.08	9.6

#### 2.1.3. Curing method

**Fig 2:**
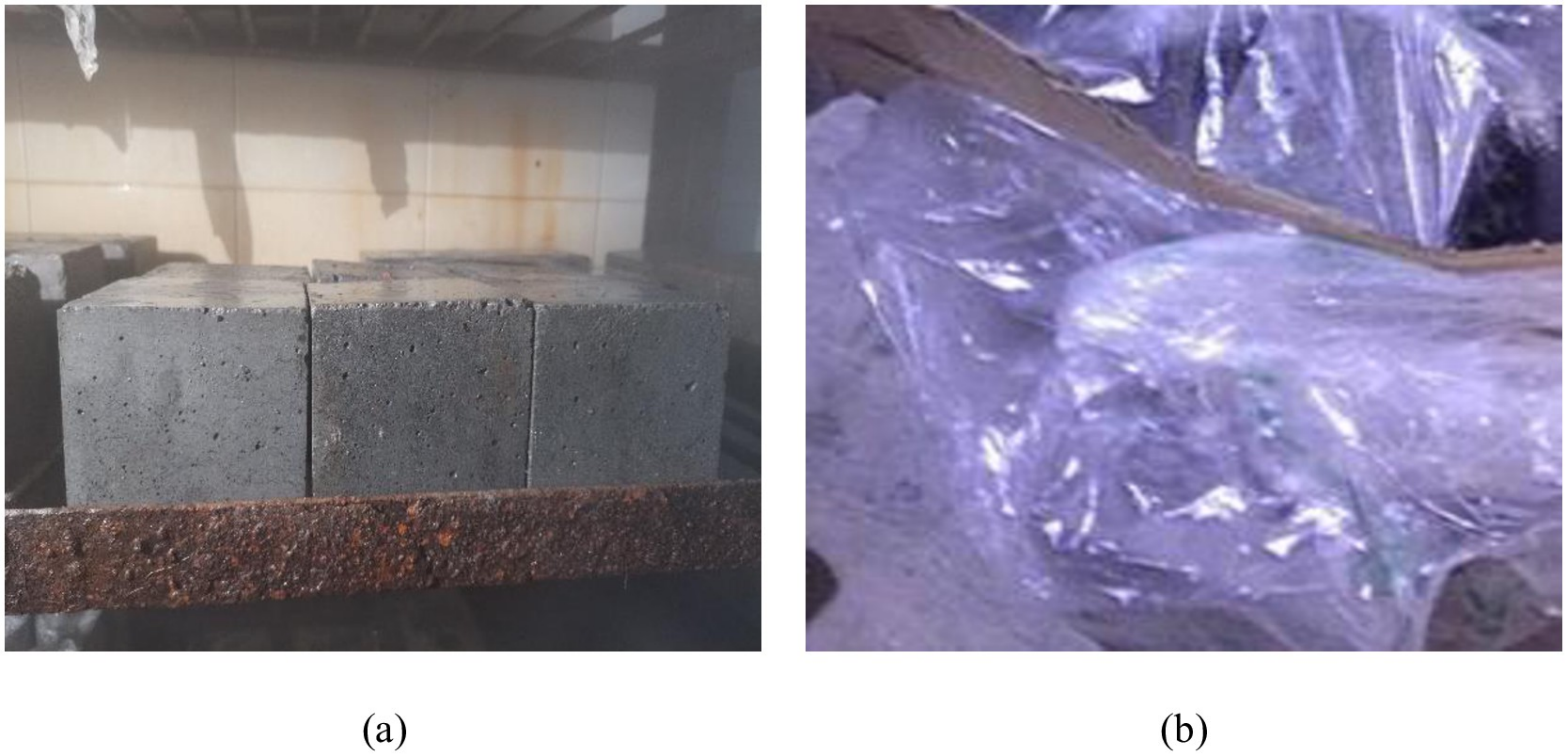
Curing method of concrete: (a) standard curing environment; (b) 5 °C curing and −3 °C curing environments.

The effects of low-temperature and negative-temperature curing conditions on the CSC and FRC are investigated in this study. Four groups of concrete, A-1–A-4, were prepared under standard curing, 5 °C curing, and −3 °C curing conditions. Tests were performed according to the Standard for Test Methods for the Performance of Ordinary Concrete Mixes (GB/T50080-2016). A day after demolding, some of the samples were placed in a standard maintenance room. Three days after demolding, the other samples were placed in an atmospheric simulation box in which the temperature and humidity were 5 ± 0.2 °C and 95%, respectively. Five days after demolding, the other samples were placed in an atmospheric simulation box in which the temperature and humidity were −3 ± 0.2 °C and 95%, respectively. Because humidity was difficult to maintain in low-temperature and negative-temperature environments, initially, the concrete specimen was positioned in an atmospheric simulation chamber. It was then sealed in a bag fitted with a hygrometer to guarantee that the humidity inside the bag consistently exceeded 90%. The curing process of concrete under the three conditions is shown in Fig 2.

### 2.2. Experimental design

#### (1) Compressive strength tests

**Fig 3:**
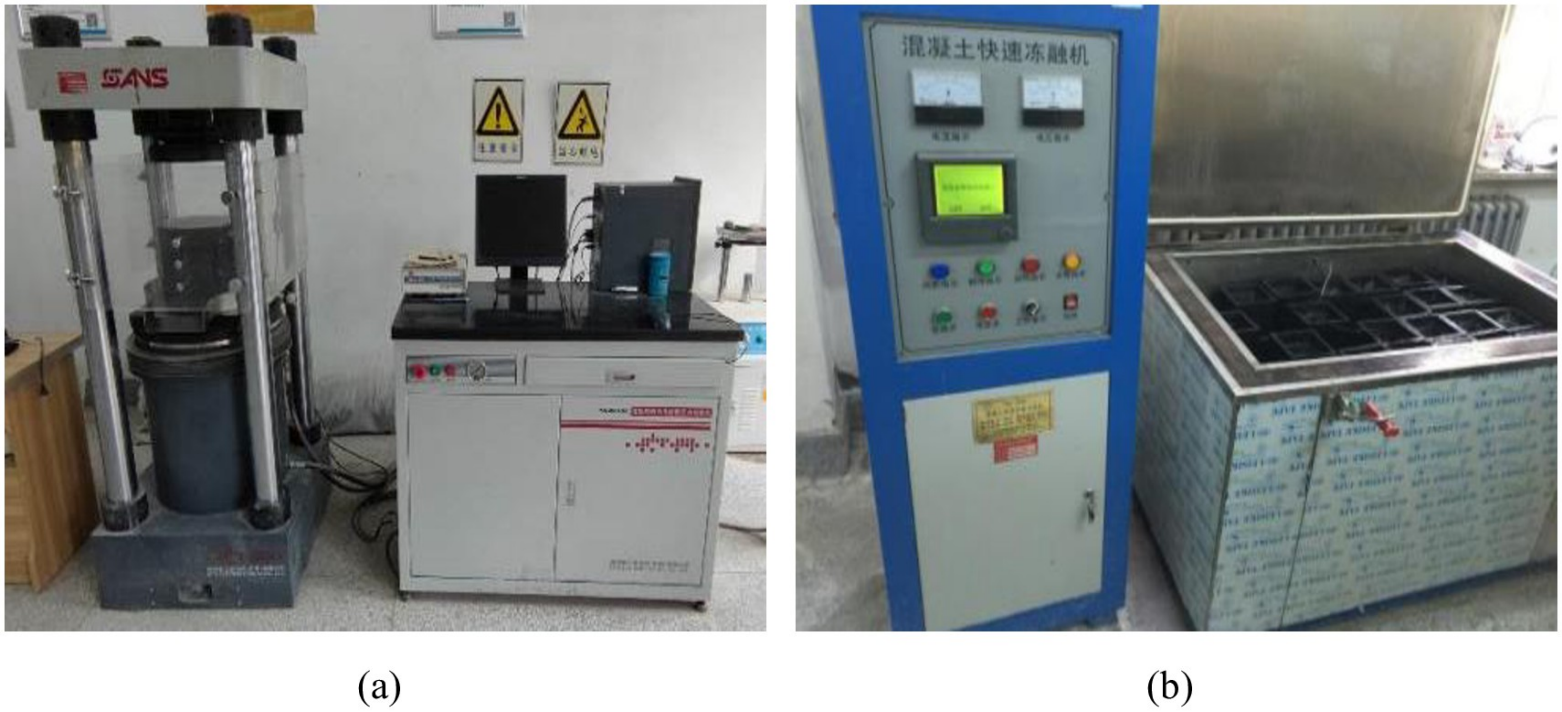
(a) Electro-hydraulic servo pressure testing machine; (b) Concrete freeze-thaw cycle testing machine.

The tests were conducted with reference to the Standard for Physical and Mechanical Properties of Concrete Test Methods (GB/T 50081–2019); the compressive strength of four groups of concrete, A-1–A-4, was tested. Test cubes (100 mm × 100 mm × 100 mm) were prepared and cured for 7, 14, 28, 56, 84, and 180-d. Mechanical performance was tested using a pressure testing machine (an electrohydraulic servo press). The test equipment is shown in Fig 3(a).

#### (2) F–T test

According to the “Quick-freezing Method” in the Standard Test Method for Long-term Performance and Durability of Ordinary Concrete (GB/T 50082–2009), the required specimen was a rectangular test block with dimensions of 100 mm ×100 mm × 400 mm. The design ages of concrete under standard curing, 5 °C curing, and −3 °C curing conditions were 28, 56, and 84-d, respectively. Four days before the concrete samples reached their design age, they were immersed in water, and a frost resistance test was implemented. Moisture was removed from the surface of samples before the test. Then, the initial masses and DEM of the specimens were measured. The FTC was completed within 4 h; the time for thawing was not less than 1/4 of the entire cycle time. The minimum and maximum temperatures at the center of the specimen were controlled within −18 ± 2 and 5 ± 2 °C, respectively. The masses and RDEM of the specimens were measured after 25 FTCs. The test was stopped when the mass loss rate (MLR) reached 5% or the RDEM reached 60% of the initial value. The test equipment is shown in Fig 3(b). To calculate the MLR, Eq (1) is used:

$$\Delta W_{N}=\frac{W_{0}-W_{N}}{W_{0}}\times 100\%$$ (1)

where Δ*W*_*N*_ represents the MLR after *N* FTCs; *W*_0_ represents the initial mass; and *W*_*N*_ represents the mass after *N* FTCs. To calculate the RDEM, Eq (2) is used:

$$P_{N}=\frac{E_{N}}{E_{0}}\times 100\%=\frac{f_{N}^{2}}{f_{0}^{2}}\times 100\%$$ (2)

where *P*_*N*_ represents the RDEM after *N* FTCs; *E*_0_ represents the initial DEM; *E*_*N*_ represents the DEM after *N* FTCs; and *f*_0_, *f*_*N*_ represent the initial transverse fundamental frequency before and after the F–T test, respectively.

#### (3) NMR test

In this study, the relaxation time method of NMR was applied to test the pore structure characteristics of concrete after F–T. A 100 mm × 100 mm × 100 mm cubic test block was used in the test. The debris on the surface of each specimen was cleaned before the test began. Then, the test block was vacuumed and pressurized with full water at a 10-MPa pressure for 24-h. Subsequently, the concrete specimen was removed and wrapped in plastic to prevent water loss. Finally, the specimens were placed in the coil of the NMR instrument for testing. The experimental equipment used was a Suzhou Niumai Macro MR12-150H-I low-field NMR instrument.

According to theory, NMR can measure the transverse relaxation time (*T*_2_) of hydrogen atoms in saturated concrete. The distribution of *T*_2_ is closely related to the distribution of pores in concrete. The distribution of water-bearing pores inside concrete can be approximated using Eq (3) [39]:

$$\frac{1}{T_{2}}=\rho_{2}\frac{S}{V}$$ (3)

where *ρ*_2_ indicates the surface relaxation rate of concrete, and *S*/*V* is the specific surface area of the pores depending on the pore shape. For spherical or columnar pores, Eq (3) can be further converted into a relationship in terms of pore radius, as given by Eq (4) [37]:

$$\frac{1}{T_{2}}=F_{S}\frac{\rho_{2}}{r}$$ (4)

where *r* is the pore radius, and *F*_*S*_ denotes the pore shape factor. In this study, the pore shape is approximated to be columnar [40], and the relaxation rate is 5 μm/s. Therefore, the relationship between pore size and *T*_2_ can be simplified as Eq (5)

$$r=0.01T_{2}$$ (5)

## 3. Experimental results and analysis

### 3.1. CSC

**Fig 4:**
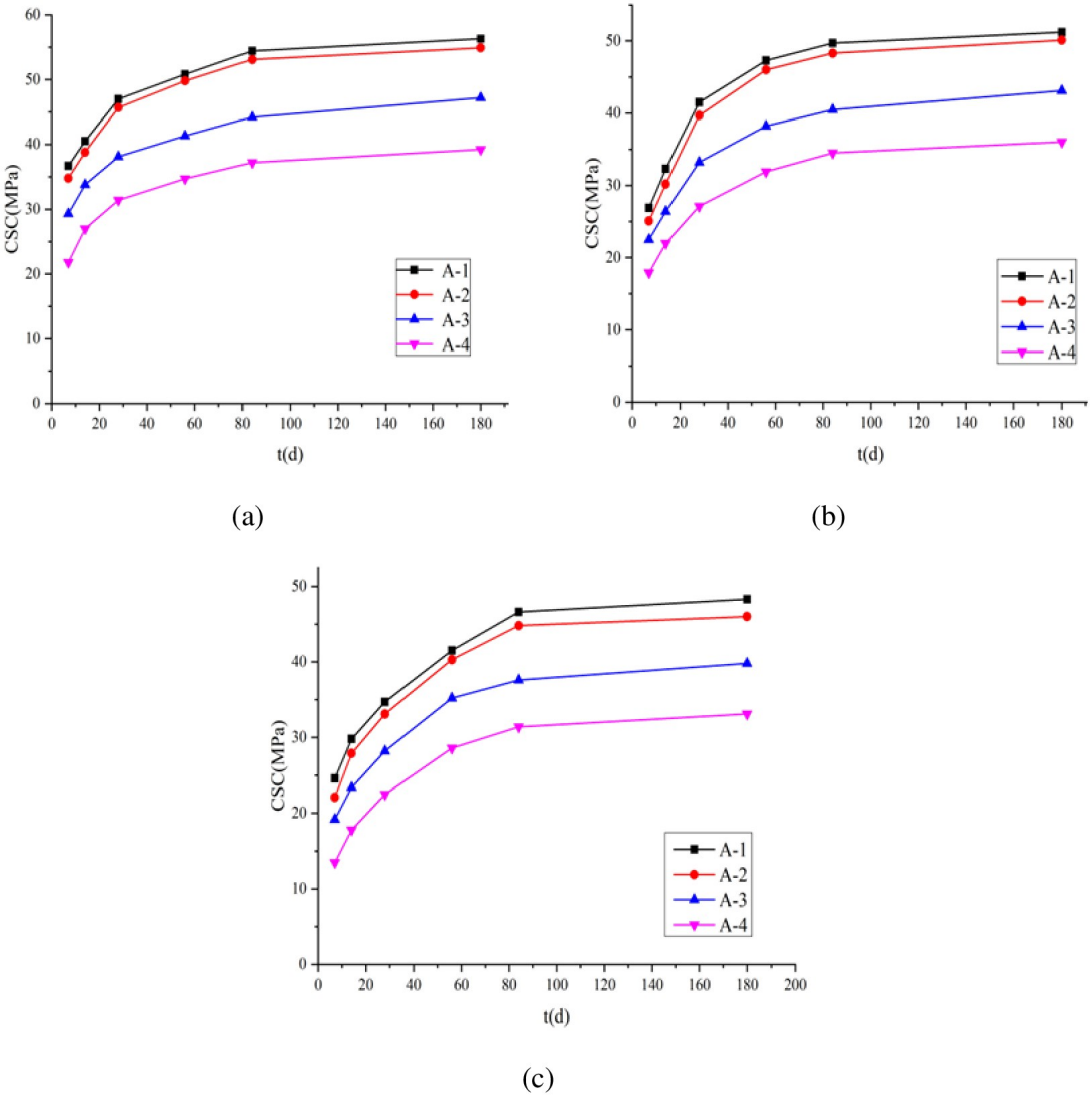
Variation of CSC with curing age under three curing conditions: (a) standard curing; (b) 5 °C curing; (c) −3 °C curing.

The variation of CSCs in groups A-1–A-4 under the standard curing, 5 °C curing, and −3 °C curing conditions is shown in Fig 4. The figure indicates that the CSC exhibited an increasing trend and then plateaus with increasing curing age. The CSC of group A-1 under standard curing conditions for 28-d was 47.0 MPa. The CSCs in groups A-2, A-3, and A-4 were 0.972, 0.811, and 0.668 times that of group A-1, respectively. The CSC of A-1 under 5 °C curing conditions for 28-d was 41.5 MPa. The CSCs of A-2, A-3, and A-4 were 0.957, 0.800, and 0.653 times that of group A-1, respectively. The CSC of A-1 after 28-d under −3 °C curing conditions was 34.7 MPa. The CSCs of A-2, A-3, and A-4 were 0.954, 0.813, and 0.648 times that of the CSC of group A-1, respectively. The compressive strengths of the four groups of concrete exhibited a decreasing trend with increasing air content under the same curing conditions. The main reason for the decrease in compressive strength is that, under the same mix proportion and curing conditions, increasing the dosage of AEA leads to an increase in the number of approximately spherical air bubbles introduced into the concrete, resulting in an increase in initial air content. These introduced air bubbles increase the porosity of the concrete, causing a reduction in the effective load-bearing capacity per unit volume of the concrete, thus resulting in a loss of concrete strength. Moreover, the CSC decreases with a decreases curing temperature. This conclusion was in agreement with Dai et al.’s findings [41]. They noted that a lower curing temperature results in a reduced hydration rate of cement within concrete. This leads to the production of fewer hydration products within the same period, ultimately resulting in diminished concrete strength.

After 28-d of standard curing, the compressive strengths of A-1–A-4 were 47, 45.7, 38.1, and 31.4 MPa, respectively (Fig 4). When cured under 5 °C curing conditions for 56 d, the compressive strengths of A-1–A-4 were 47.3, 46, 38.2, and 31.9 MPa, respectively. After curing under −3 °C curing conditions for 84 d, the CSCs of A-1–A-4 were 46.6, 44.8, 37.6, and 31.4 MPa, respectively; Clearly, a lower curing temperature increases the time needed for concrete to reach its standard 28-d compressive strength, demonstrating a noticeable age lag phenomenon. This is mainly because the formation of concrete strength relies primarily on the hydration reaction of cement. Low or negative temperature curing reduces the hydration reaction rate of cement. However, as the curing period progresses, concrete continues to undergo hydration. Therefore, the strength of concrete under low or negative temperature curing conditions can reach the strength of concrete under standard curing conditions. (The CSC of concrete under 5 °C curing and −3 °C curing conditions can exceed or approach the CSC under standard curing conditions by increasing the curing age.) Therefore, to achieve accurate comparison, the FRC values under the standard curing, 5 °C curing, and −3 °C curing conditions for 28, 56, and 84-d, respectively, were compared.

### 3.2. MLR

**Fig 5:**
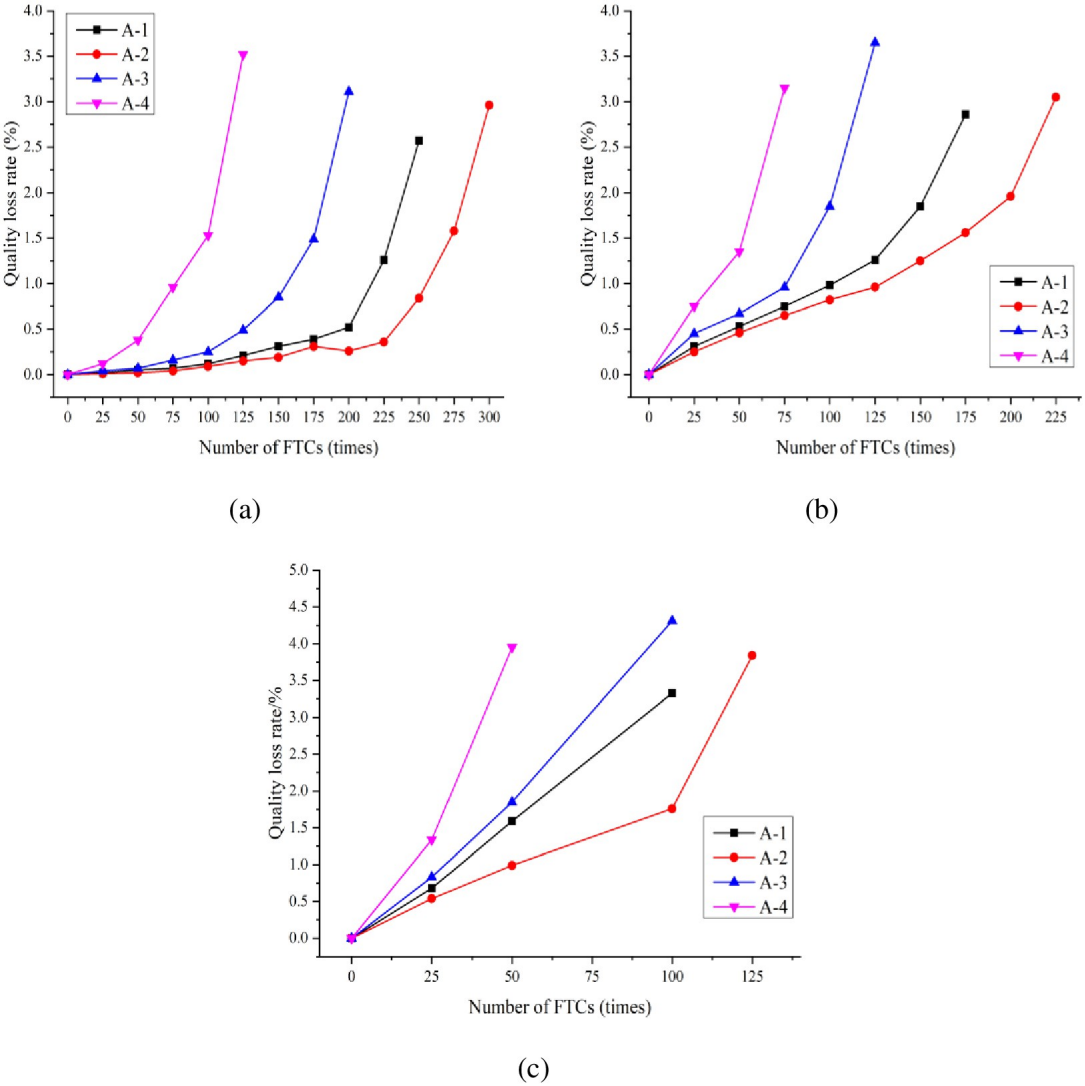
Variation of MLR of concrete under three curing conditions after F–T: (a) standard curing; (b) 5 °C curing; (c) −3 °C curing.

The MLR reflects the degree of spalling and moisture absorption of a concrete surface. The variation law of the MLR of concrete under the standard curing, 5 °C curing, and −3 °C curing conditions is shown in Fig 5. In the figure, the MLR of concrete under the three curing conditions tends to increase with the number of FTCs. Under standard curing conditions, after 250 FTCs, the MLRs of A-1 and A-2 were 2.57% and 0.84%, respectively. After 200 FTCs, the MLR of A-3 reached 3.11%. After 125 FTCs, the MLR of A-4 reached 3.52%. Evidently, the change range of the MLRs of A-3 and A-4 exceeds those of A-1 and A-2. In terms of MLR, the FRC shows an increasing and then a decreasing trend with increasing air content; the FRC is optimal when the air content was 3.6%.

Under standard curing conditions, the MLR of A-2 concrete was 0.84% after 250 FTCs. Under 5 °C curing conditions, the MLR of A-2 reached 3.05% after 225 FTCs. Under −3 °C curing conditions, the MLR of A-2 reached 3.84% after 100 FTCs. Evidently, the lower the curing temperature, the faster the increase in the MLR of concrete.

Dai et al. [41] through the study of FTD of concrete under −3 °C curing conditions, showed that when the water-to-cement ratio was the same, the number of F-T cycles of concrete specimens of the same strength under −3 °C curing conditions to reach the damage was lower than that of the standard curing, and the time of the damage was earlier than that of the standard curing. It can be seen that by prolonging the curing age of −3°C concrete, the strength of concrete can reach the strength of standard curing at 28-d age, but the concrete that reaches the same strength can not achieve the same frost durability. This is similar to the results of this paper. By studying the effect of different curing temperatures (5 °C, 20 °C, 50 °C) on the F-T resistance of hydraulic concrete, Jin et al. [42] found that the highest MLR of concrete was observed at a curing temperature of 5 °C, which shows that as the curing temperature decreases, the greater the change in the MLR of the concrete, and the poorer the concrete’s frost resistance. Unlike the present study, the curing temperature was 50 °C in the study of jin et al. [42] while it was −3 °C in this paper. Chen et al. [43] investigated the relationship between curing conditions and FRC and found that after the number of F-T cycles of concrete under curing conditions of 3 °C and 10 °C reached 175 and 225 times, respectively, the FRC was significantly reduced and the surface appeared to be severely damaged. In contrast, when the number of F-T cycles of concrete specimens under 20 °C curing condition reached 300 times, the specimens were basically intact. This phenomenon is consistent with the pattern presented in this study, with the lowering of the curing temperature, the worse the FRC.

### 3.3. RDEM

**Fig 6:**
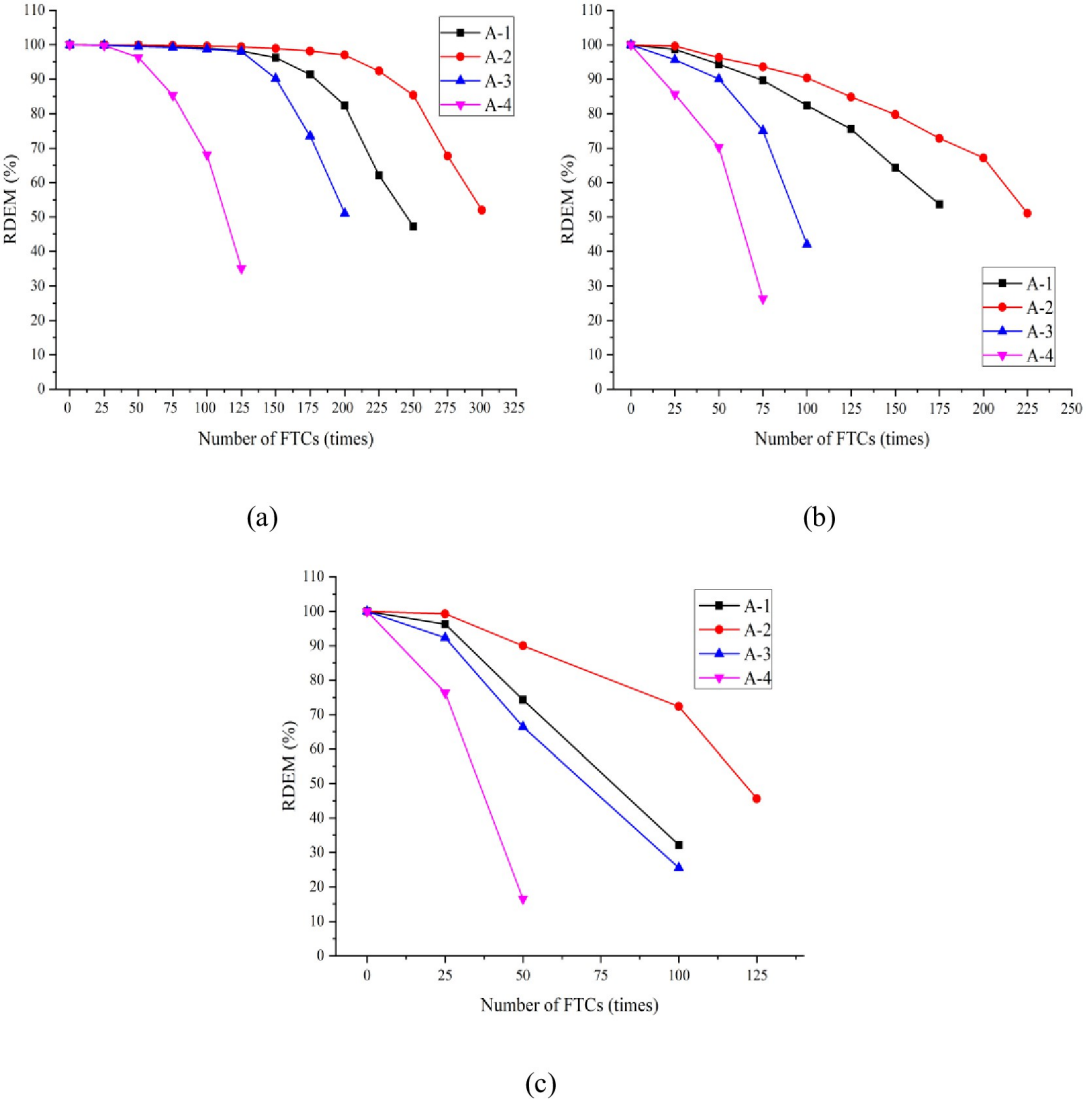
Variation of RDEM of concrete under three curing conditions after F–T: (a) SC; (b) 5 °C curing; (c) −3 °C curing.

The RDEM reflects the development of cracks and pores inside the concrete and the damage to concrete under the action of F–T. The variation law of the RDEM of concrete under the standard curing, 5 °C curing, and −3 °C curing conditions is shown in Fig 6. The figure indicates that the RDEM of concrete under the three curing conditions tends to decrease with the increasing number of FTCs. However, the decreasing trends vary considerably. When the number of FTCs was the same, the RDEM of the four groups of concrete decreases with increasing air content. In particular, the RDEM of A-4 linearly decreases with the increasing number of FTCs; the range of decrease was broad. The failure criteria were reached relatively quickly, indicating inadequate frost resistance.

For groups A-1–A-4 under standard curing conditions and subjected to 225, 275, 175, and 100 FTCs, the RDEM decayed to less than 60%, respectively. As shown in Fig 5(a), the change range of RDEM values of A-1 and A-2 was small. The RDEM of group A-4 exceeds those of groups A-1 and A-2. Among the four groups of concrete, A-2 can withstand the highest number of FTCs before satisfying the failure criteria (i.e., it has the best frost resistance performance). Both insufficient and excessive use of AEA negatively impact the RFC.

Dai et al. [41] have confirmed this finding. After 150, 200, 100, and 50 FTCs, the RDEM values of concrete groups A-1–A-4 under 5 °C curing conditions decayed to less than 60%. After 50, 75, 50, and 25 FTCs, the RDEM values of groups A-1–A-4 of concrete under −3 °C curing conditions decayed to less than 60%. Evidently, the number of FTCs corresponding to the decay of the RDEM to less than 60% for all four groups of concrete under 5 °C curing and −3 °C curing conditions was reduced to some extent compared with that under standard curing conditions. The number of FTCs in group A-2 under −3 °C curing conditions was only 1/4 of that under standard curing conditions. Therefore, the lower the curing temperature, the lower the number of FTCs required to satisfy the failure criteria, that is, low-temperature and negative-temperature curing accelerates the FTD to concrete.

### 3.4. Pore structure

#### 3.4.1. T_2_ spectral distribution.

**Fig 7:**
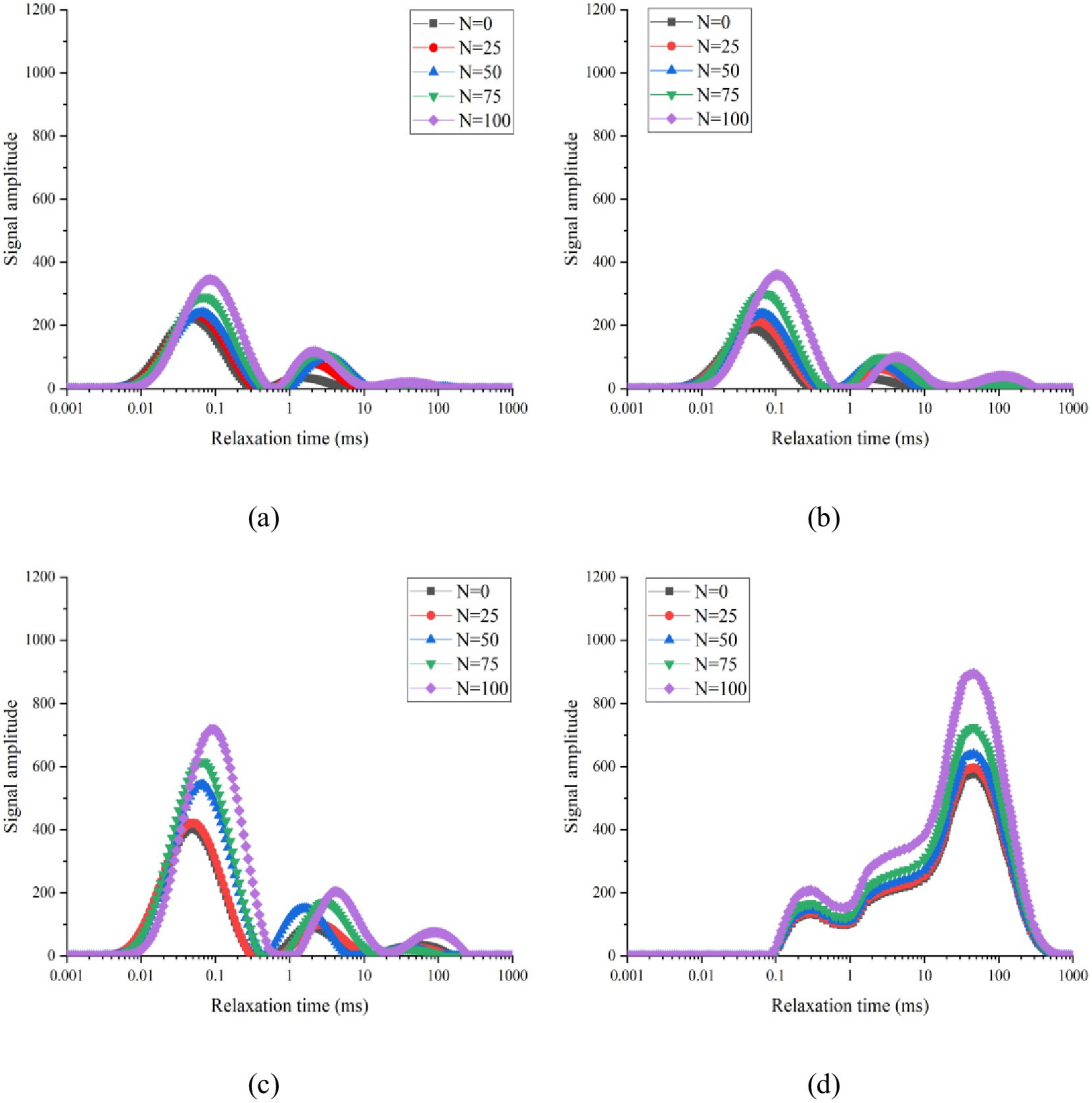
Variation of T_2_ spectrum of 0.38 water–binder ratio of concrete during F–T process under standard curing conditions: (a) Air content = 1.5%; (b) Air content = 3.6%; (c) Air content = 6.4%; (d) Air content = 9.6%.

**Fig 8:**
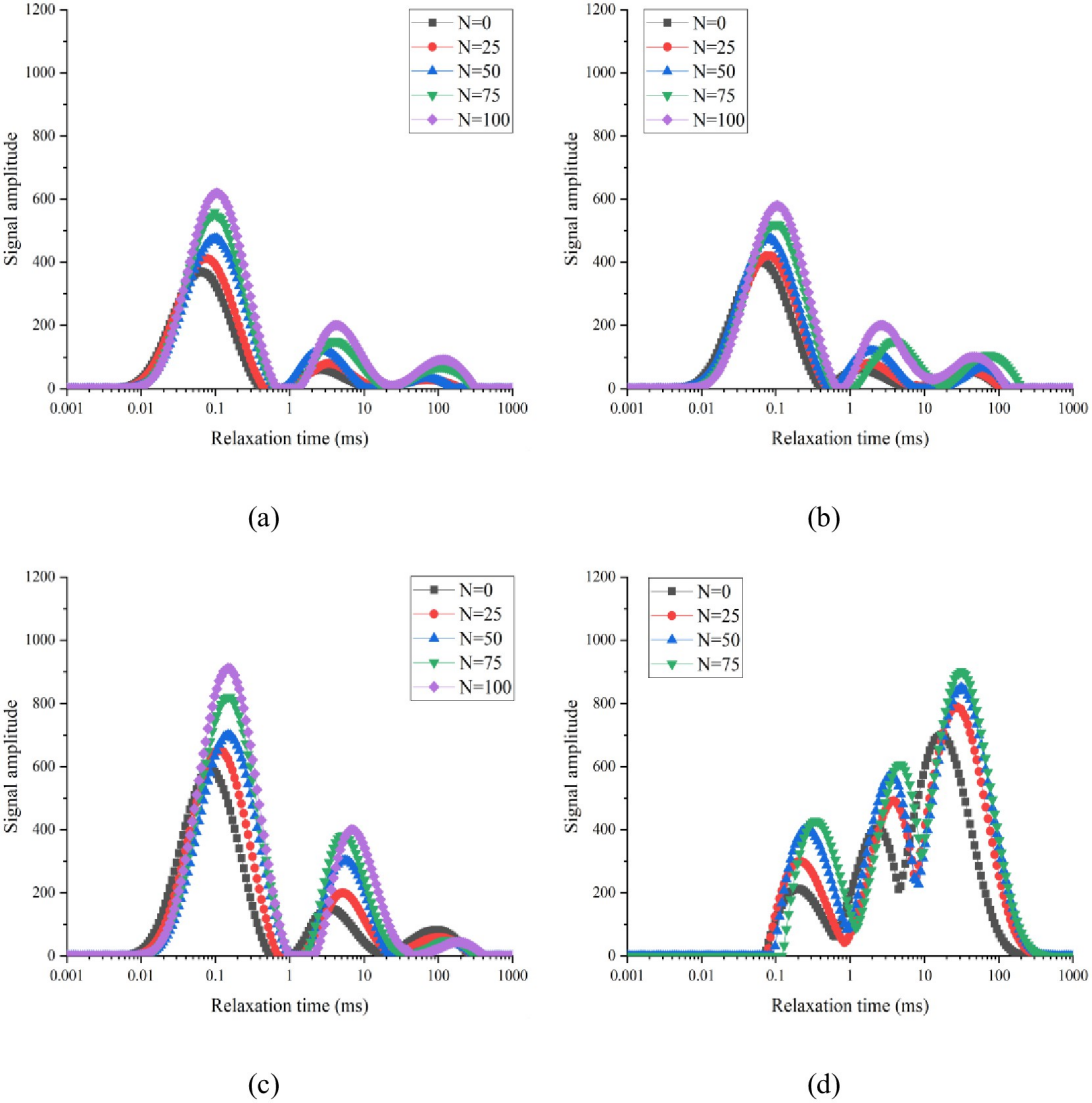
Variation of T_2_ spectrum of 0.38 water–binder ratio of concrete during F–T process under 5 °C curing conditions: (a) Air content = 1.5%; (b) Air content = 3.6%; (c) Air content = 6.4%; (d) Air content = 9.6%.

**Fig 9:**
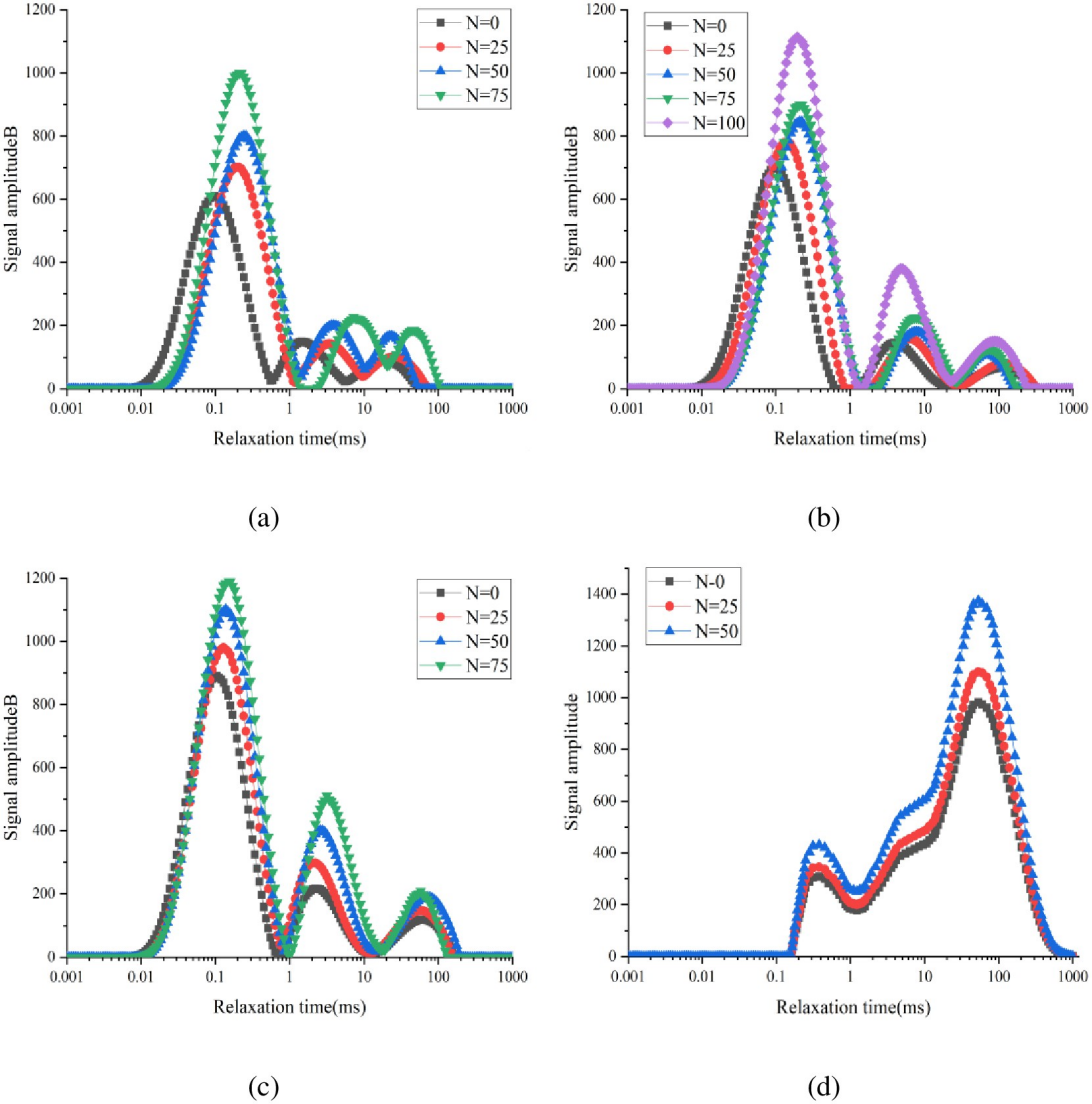
Variation of T_2_ spectrum of 0.38 water–binder ratio of concrete during F–T process under −3 °C curing conditions: (a) Air content = 1.5%; (b) Air content = 3.6%; (c) Air content = 6.4%; (d) Air content = 9.6%.

The T_2_ spectral distribution is mainly related to the nature of hydrogen protons inside concrete and reflects the pore structure characteristics. The vertical signal amplitude of the T_2_ spectral curve reflects the number of concrete pores. The larger the ordinate, the greater the number of pores present. The x coordinate represents the relaxation time; the longer the relaxation time, the larger the pore size of the sample. The variation law of the T_2_ spectra of the four groups of concrete under the standard curing, 5 °C curing, and −3 °C curing conditions is shown in Figs 7–9, respectively.

After 0, 25, 50, 75, and 100 FTCs, the main peaks of the T_2_ spectral curves of group A-2 under standard curing conditions were 221.667, 231.051, 239.831, 290.136, and 345.253, respectively. After 0, 25, 50, 75, and 100 FTCs, the main peaks of the T_2_ spectral curves of group A-2 under 5 °C curing conditions were 400.216, 420.224, 475.350, 520.275, and 580.000, respectively. After 0, 25, 50, and 75 FTCs, the main peaks of the T_2_ spectral curves of group A-2 under −3 °C curing conditions were 700.681, 779.480, 846.123, and 1113.717, respectively. When the air content was the same, the main peak of the T_2_ spectral curve of concrete increases as the curing temperature decreases (i.e., porosity increases).

As shown in Fig 7, the T_2_ spectral curve of groups A-1–A-4 under standard curing conditions presents one main peak and two secondary peaks. The main peaks of groups A-1, A-2, and A-3 appeared at short relaxation times, whereas the second peak appeared at long relaxation times. For group A-4, the main peak appeared at a long relaxation time, whereas the secondary peak appeared at a short relaxation time. Under the same curing condition, the pore size inside the concrete increased with the air content. After 0, 25, 50, 75, and 100 FTCs, the relaxation times corresponding to the main peaks of group A-1 were 0.0488ms, 0.0523ms, 0.0644ms, 0.0691ms, and 0.1047ms, respectively. The T_2_ spectral curve was observed to move to the right as the number of FTCs increased. Hence, the internal pores of concrete gradually increase in size with the number of FTCs. This is because under the action of F–T fatigue stress, the saturated water inside the concrete produces frost heave stress, leading to the development of a concrete aperture in the direction of a large hole. In this study, an NMR measurement instrument was used to convert the obtained T_2_ spectra into porosity. Subsequently, the proportion of different pore sizes in the total porosity was determined. Accordingly, the FRC was evaluated from a microscopic perspective.

#### 3.4.2. Porosity variation

**Fig 10:**
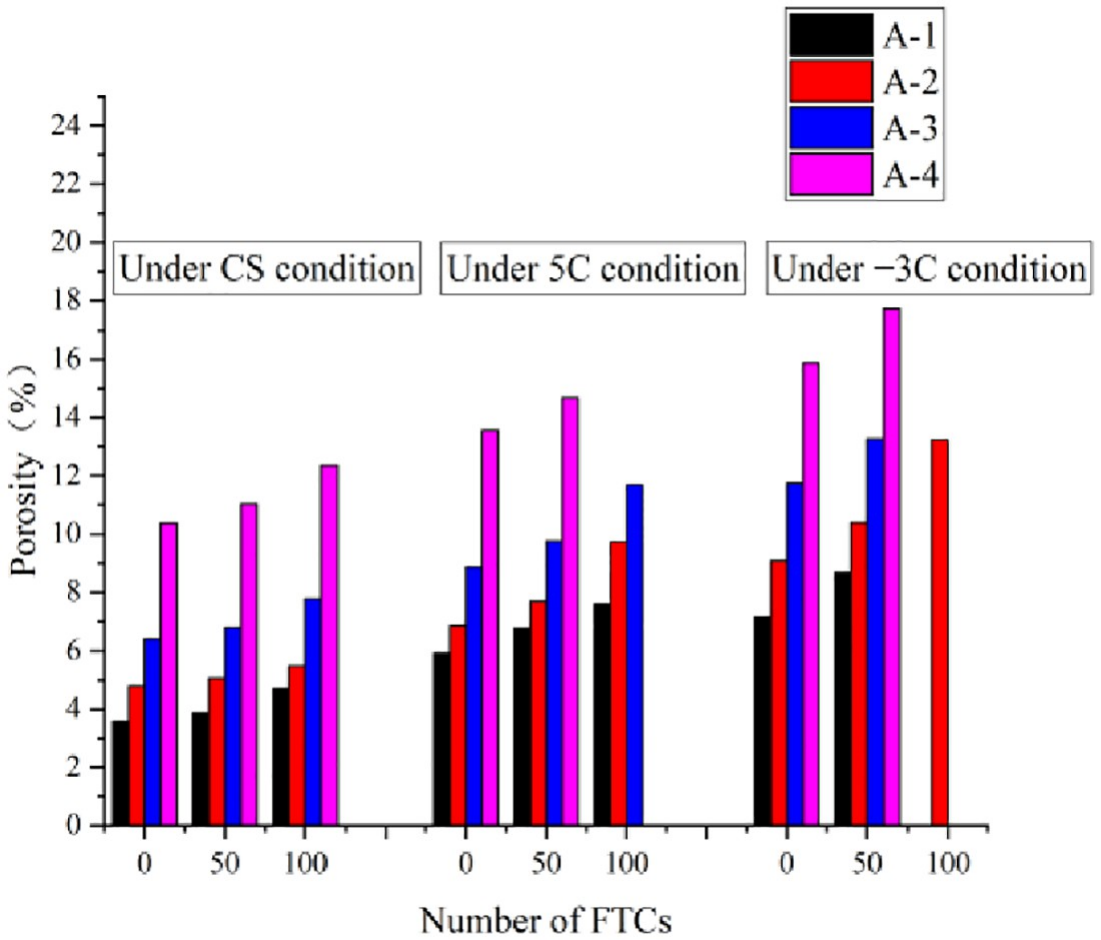
Porosity changes in concrete after F–T under three curing conditions.

The variation in concrete porosity in groups A-1–A-4 under the three curing conditions is shown in Fig 10. (Because the three concrete groups A-1, A-2 and A-3 meet the failure criteria 100 times before, only the data for the A-2 concrete group are available.) The figure indicates that the porosities of A-1–A-4 without F–T under standard curing conditions are 3.58%, 4.79%, 6.42%, and 10.38%, respectively. After 100 FTCs, the porosities of groups A-1–A-4 were 4.71%, 5.48%, 7.78%, and 12.36%, increasing by 1.13%, 0.69%, 1.36%, and 1.98%, respectively. Zhang et al. [44] examined the effects of freeze-thaw cycles on concrete’s mechanical properties and microstructure, focusing on samples with two distinct water-cement ratios. Initially, they found that concrete porosity showed little change. Yet, from the 50th to the 125th cycle, a marked increase in NMR porosity was noted. These findings mirror the pore variation trends we observed in our research. Under the same curing conditions, the porosity of concrete tends to increase with the number of FTCs. The main reason for this phenomenon is that during the FTC of concrete, the saturated water contained in the internal pores undergoes freezing and expansion under the fatigue environment of the FTC, leading to an increase in the size and quantity of pores inside the concrete. This results in a continuous increase in the porosity of the concrete and the formation of interconnected pores and microcracks in the surrounding pores. As a result, the porosity of the concrete tends to increase after the FTC. Luo et al. [45] noted a comparable pattern, discovering that the increase in freeze-thaw cycles led to a progressive increase in micropores and cracks within the concrete. Over time, these small imperfections accumulated, ultimately transforming into major macro defects.

Under the same curing conditions, as the F–T progressed, the porosity of group A-2 had the least increase, corresponding to the optimal freezing resistance of this concrete group. Therefore, the air content range was reasonable. When the air content was extremely low, the effect of improving the FRC was not evident. In contrast, when the introduced air content was overly high, the FRC decreased. This is because the addition of AEA can create more closed pores in the concrete. These closed pores effectively block the pathways for moisture to enter the interior of the concrete, making it difficult for moisture to penetrate the concrete. As a result, the damage caused by water freezing and expanding inside the concrete is reduced. However, excessive addition of air entraining agents in the concrete results in excessively high initial air content and porosity in the hardened concrete. The excessive number of pores, along with the interconnected pores, not only reduces the strength of the concrete but also decreases its frost resistance. From the above analysis, it can be concluded that an appropriate dosage of AEA can reduce the damaging effects of F–T on concrete, thereby effectively improving its frost resistance. As shown in Fig 8, the porosities of concrete group A-2 under the standard curing, 5 °C curing, and −3 °C curing conditions, without F–T were 4.79%, 7.82%, and 10.39%, respectively. When the air content was the same, the porosity of concrete tended to increase with decreasing curing temperature. Maciej et al. [4] found that high temperatures help reduce concrete porosity, suggesting increased porosity with low-temperature curing. In contrast, our study utilized CO_2_ curing, differing from their methodology.

**Fig 11:**
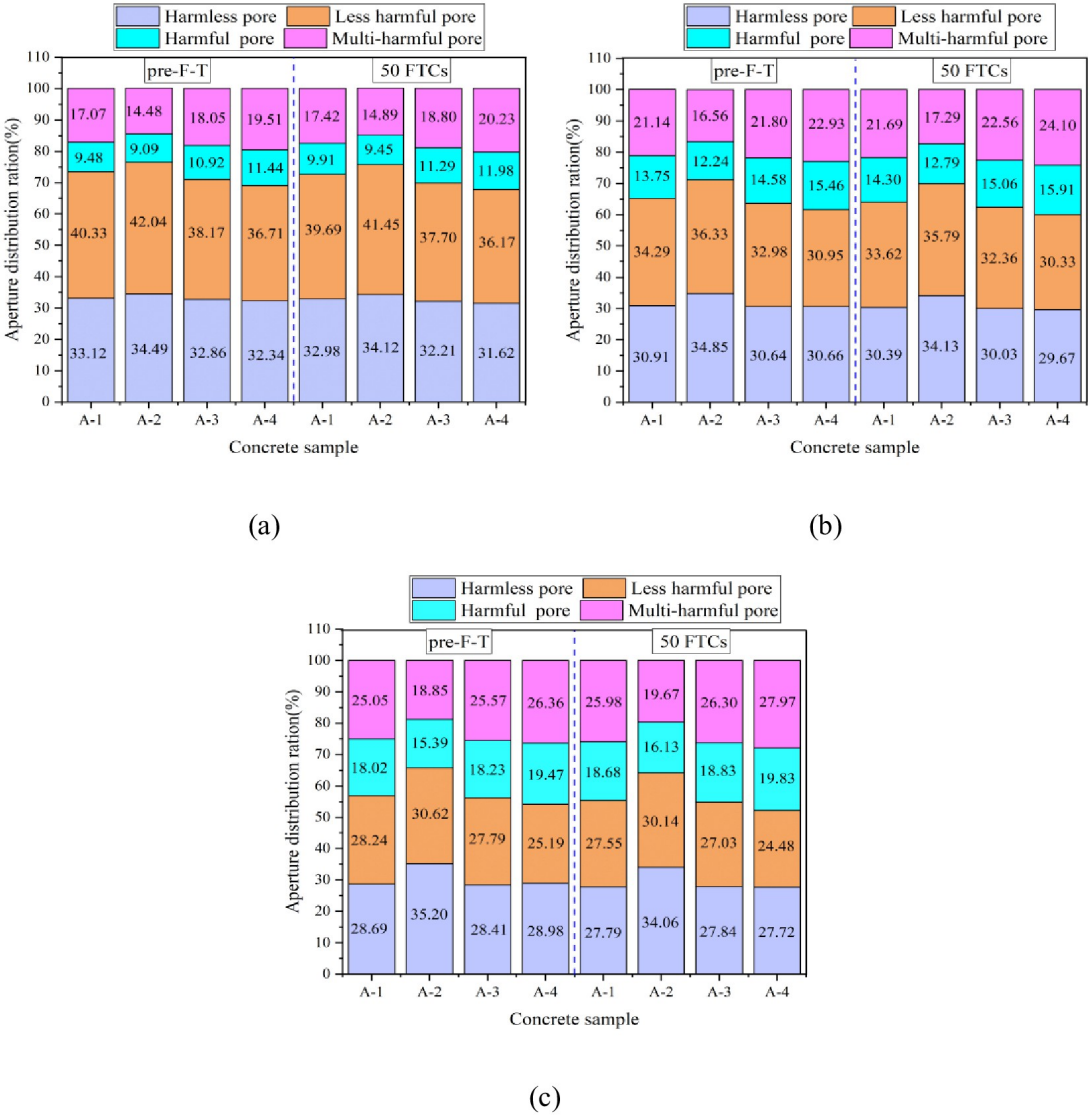
NMR aperture distribution of samples before and after 100 FTCs under three curing conditions: (a) standard curing; (b) 5 °C curing; (c) −3 °C curing.

According to the pore structure division theory proposed by Wu et al. [46], pores can be classified into four categories: harmless pores (r < 20 nm), less harmful pores (20 nm ≤ r < 100 nm), harmful pores (100 nm ≤ r < 200 nm), and multi-harmful pores (r ≥ 200 nm). The pore size distribution ratios of concrete groups A-1–A-4 under the three curing conditions without F–T and after 50 FTCs are shown in Fig 11.

The figure shows that under the standard curing condition without F–T, the proportions of harmless, less harmful, harmful, and multi-harmful pores in group A-2 were 34.49%, 42.04%, 9.09%, and 14.48%, respectively. After 50 FTCs, the proportions of harmless, less harmful, harmful, and multi-harmful pores in this group were 34.21%, 41.45%, 9.45%, and 14.89%, respectively. This indicates that as the number of FTCs increases, the proportions of harmless and less harmful pores decrease, whereas the proportions of harmful and multi-harmful pores increase. This conclusion diverges slightly from Bai et al.’s [22] research, which focused on the frost resistance of concrete mixed with different proportions of aeolian sand. Their study found that after 75 freeze-thaw cycles, there was a noticeable overall increase in the pore size distribution across four pore types in each sample, though the degree varied. Specifically, large pores exhibited the most significant increase in porosity, whereas capillary and transition pores experienced a lesser increase, and gel pores showed minimal change. This trend could likely be due to the addition of aeolian sand, which may lead to an increase in various pore types after undergoing freeze and thaw cycles. The descending order of the four concrete groups under the three curing conditions in terms of the total percentages of harmless and less harmful pores is A-2, A-1, A-3, and A-4. This indicates that an appropriate air content can improve the internal pore structure of concrete and the FRC. By investigating the effect of dredged sand replacement rate on the FTD of concrete, Bu et al. [47] found that when the dredged sand replacement rate was 50%, the concrete had the highest percentage of harmless pores and less harmful pores, which indicated that the addition of dredged sand altered the internal structure of the concrete and improved the FRC. In contrast, in this study, the FRC was improved by adding appropriate amount of air-entraining agent to introduce tiny air bubbles.

As shown in Fig 11, the proportions of harmful and multi-harmful pores of concrete in group A-1 under standard curing conditions without F–T were 9.48% and 17.07%, respectively. After 50 FTCs, the proportions of harmful and multi-harmful pores were 9.91% and 17.42%, respectively. The proportions of harmful pores and multi-harmful pores in group A-1 under the 5 °C curing condition without F–T were 13.75% and 21.14%, respectively. After 50 FTCs, the occupancies of harmful and multi-harmful pores were 14.30% and 21.69%, respectively. The percentages of harmful and multi-harmful pores of concrete in group A-1 under −3 °C curing conditions without F–T were 18.02% and 25.05%, respectively. After 50 FTCs, the proportions of harmful and multi-harmful pores were 18.68% and 25.98%, respectively. Hence, when the curing temperature decreased, the percentage of harmful and multi-harmful pores in concrete increased; however, the FRC decreased. Jin et al. [42] by studying the effect of different curing temperatures (5 °C, 20 °C, 50 °C) on the F-T resistance of hydraulic concrete found that, after 200 times of F-T cycles, the percentage of harmful holes and multi-harmful holes in the concrete at 28-d under the curing condition of 5 °C was the largest, which indicated that, with the decrease of curing temperatures, F-T cycles resulted in the largest number of defects in the concrete under the curing condition of 5 °C, which had the poorest freezing resistance. Ma et al. [48] investigated the pore size distribution and microstructural characteristics of metakaolin and metakaolin-basalt fiber-modified concrete specimens at different curing temperatures, and compared with the standard curing environment, the total porosity and the harmful pore occupancy ratio were significantly increased at the negative curing temperature (−10 °C) due to the lower degree of hydration, which led to a decrease in the FRC. From the above two literatures, it can be seen that the percentage of harmful pores inside the concrete increases with the decrease of the curing temperature, and the freezing resistance of the concrete decreases accordingly, and this conclusion is the same as that of the present study.

## 4. Evolution analysis of concrete FTD

To describe the damage state of concrete under the action of F–T quantitatively, the RDEM was selected as the damage variable in this study. The concrete FTD factor, *D*_*N*_, for an F–T is defined by Eq (6):

$$D_{N}=1-P_{N}$$ (6)

### 4.1. FTDM of concrete

Bai et al. [22] regarded the FTD and deterioration process of concrete as irreversible energy dissipation processes of the internal microstructure of concrete under repeated F–T loading and unloading cycles. According to the fundamental principles of irreversible thermodynamics and continuum damage mechanics, the FTDM of concrete is given by Eq (7):

$$D=0.4\times [1-{\left(1-\frac{N}{N_{f}}\right)}^{\frac{1}{2k+2}}]$$ (7)

where *N*_*f*_ is the critical F–T fatigue life of concrete, and k is the material parameter.

Pei et al. [23] proposed an FTDM for concrete based on the Chaboche fatigue damage theory. The model is given by Eq (8):

$$D=1-{\left[1-\beta_{2}N(1-\beta_{1})\right]}^{\frac{1}{1-\beta_{1}}}(\beta_{1}\neq 1)$$ (8)

where *β*_1_ and *β*_2_ are material parameters.

**Fig 12:**
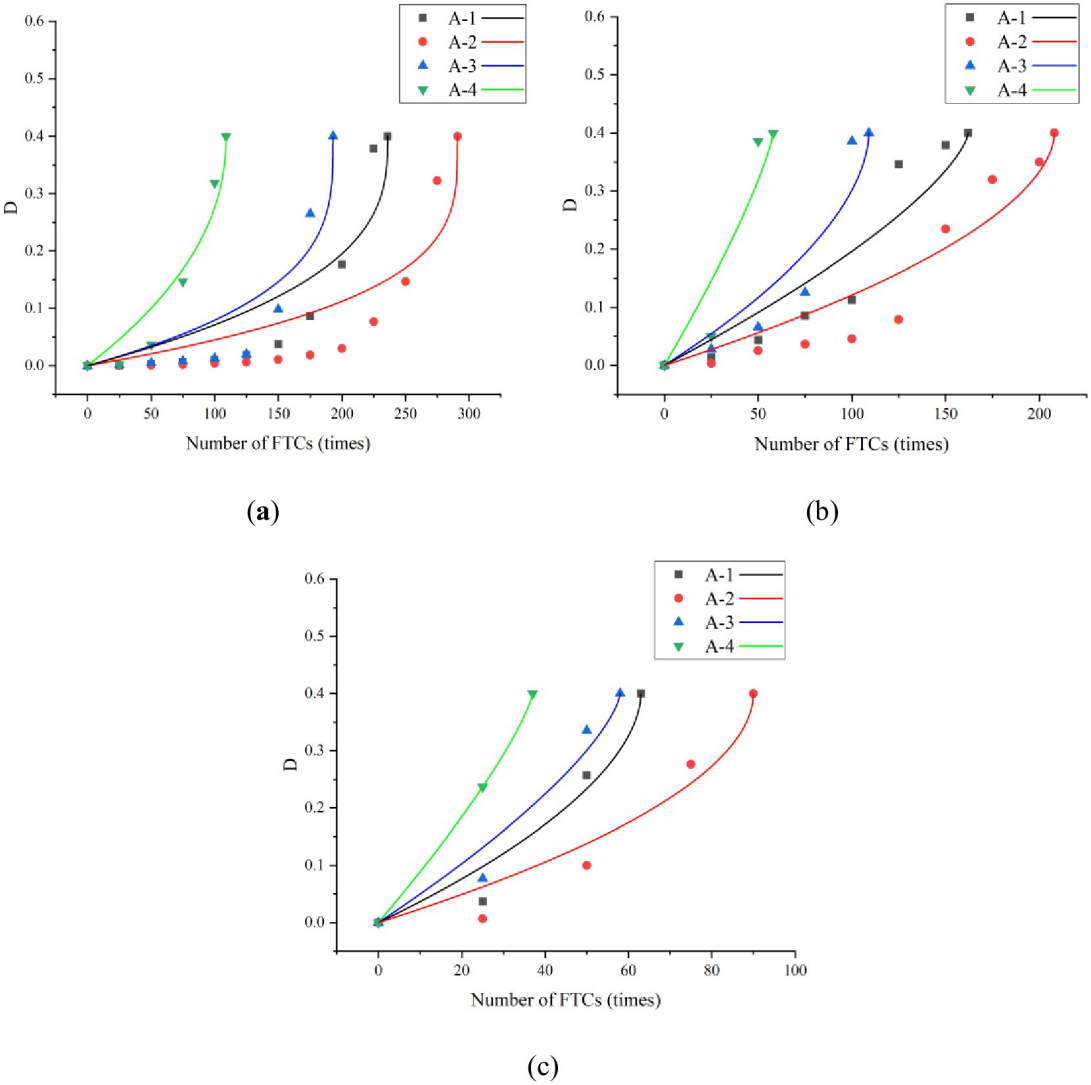
Relationship curves fitted by Bai model: (a) standard curing; (b) 5 °C curing; (c) −3 °C.

**Fig 13:**
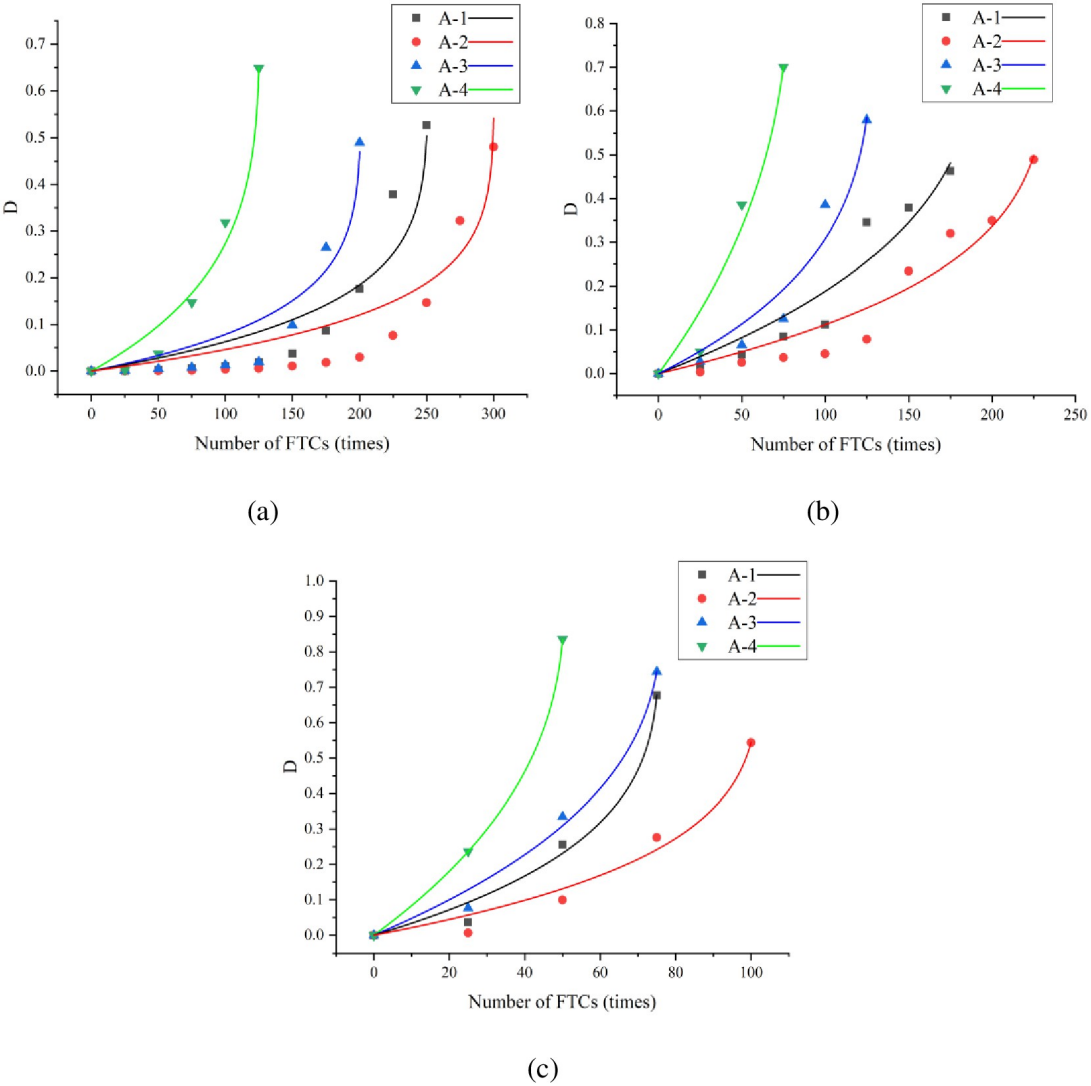
Relationship curves fitted by Pei model: (a) standard curing; (b) 5 °C curing; (c) −3 °C.

To describe the damage state of concrete accurately under the action of F–T, the FTD factor of concrete under the action of F–T is calculated using Eq (6). The Bai and Pei models were used to fit the FTD of the four groups of concrete under the three curing conditions. The parameter values and corresponding correlation coefficients of the Bai model fitting curves are shown in Fig 12 and Table 6, respectively. The parameter values and corresponding correlation coefficients of the Bai model fitting curves are shown in Fig 13 and Table 7, respectively.

**Table 6:** Relevant parameters fitted by Bai model.

Curing condition	Group	1/(2k+2)	k	*N*_*f*_	*R* ^2^	Reduced χ2
standard curing	A-1	0.356	0.405	236	0.839	0.0041
	A-2	0.283	0.767	291	0.831	0.0032
	A-3	0.303	0.650	193	0.851	0.0031
	A-4	0.469	0.066	109	0.931	0.0021
5 °C curing	A-1	0.701	−0.287	162	0.889	0.0038
	A-2	0.551	−0.093	208	0.904	0.0027
	A-3	0.562	−0.110	109	0.908	0.0037
	A-4	0.804	−0.378	58	0.899	0.0069
−3 °C curing	A-1	0.555	−0.099	63	0.960	0.00214
	A-2	0.522	−0.042	90	0.954	0.00189
	A-3	0.703	−0.289	58	0.964	0.00206
	A-4	0.796	−0.372	37	1.000	0

**Table 7:** Relevant parameters fitted by Pei model.

Curing condition	Group	*β*_1_	*β*_2_	*R* ^2^	Reduced χ2
standard curing	A-1	5.105 × 10^−4^	−6.803	0.893	0.0038
	A-2	3.897 × 10^−4^	−7.54	0.848	0.0037
	A-3	5.926 × 10^−4^	−7.397	0.909	0.0030
	A-4	1.600 × 10^−3^	−3.969	0.976	0.0019
5 °C curing	A-1	1.490 × 10^−3^	−2.444	0.919	0.0032
	A-2	5.430 × 10^−3^	−2.895	0.934	0.0023
	A-3	1.900 × 10^−3^	−3.088	0.951	0.0033
	A-4	5.160 × 10^−3^	−1.454	0.964	0.0057
−3 °C curing	A-1	3.220 × 10^−3^	−3.097	0.987	0.0019
	A-2	2.060 × 10^−3^	−3.749	0.978	0.0015
	A-3	4.570 × 10^−3^	−1.861	0.989	0.0017
	A-4	7.830 × 10^−3^	−1.526	1.000	0

The fitting results indicate that the fitted curves of both models approximate the experimental results and have high correlation coefficients. The correlation coefficient ranges for the Bai and Pei model fitted curves are 0.83–1 and 0.89–1, respectively. The Pei model can predict the evolution of concrete FTD more accurately. In particular, the FTD process of concrete under −3 °C curing conditions was fitted more accurately, and the correlation coefficients, R^2^, exceeded 0.97.

### 4.2. FTDM of equal-strength concrete

**Fig 14:**
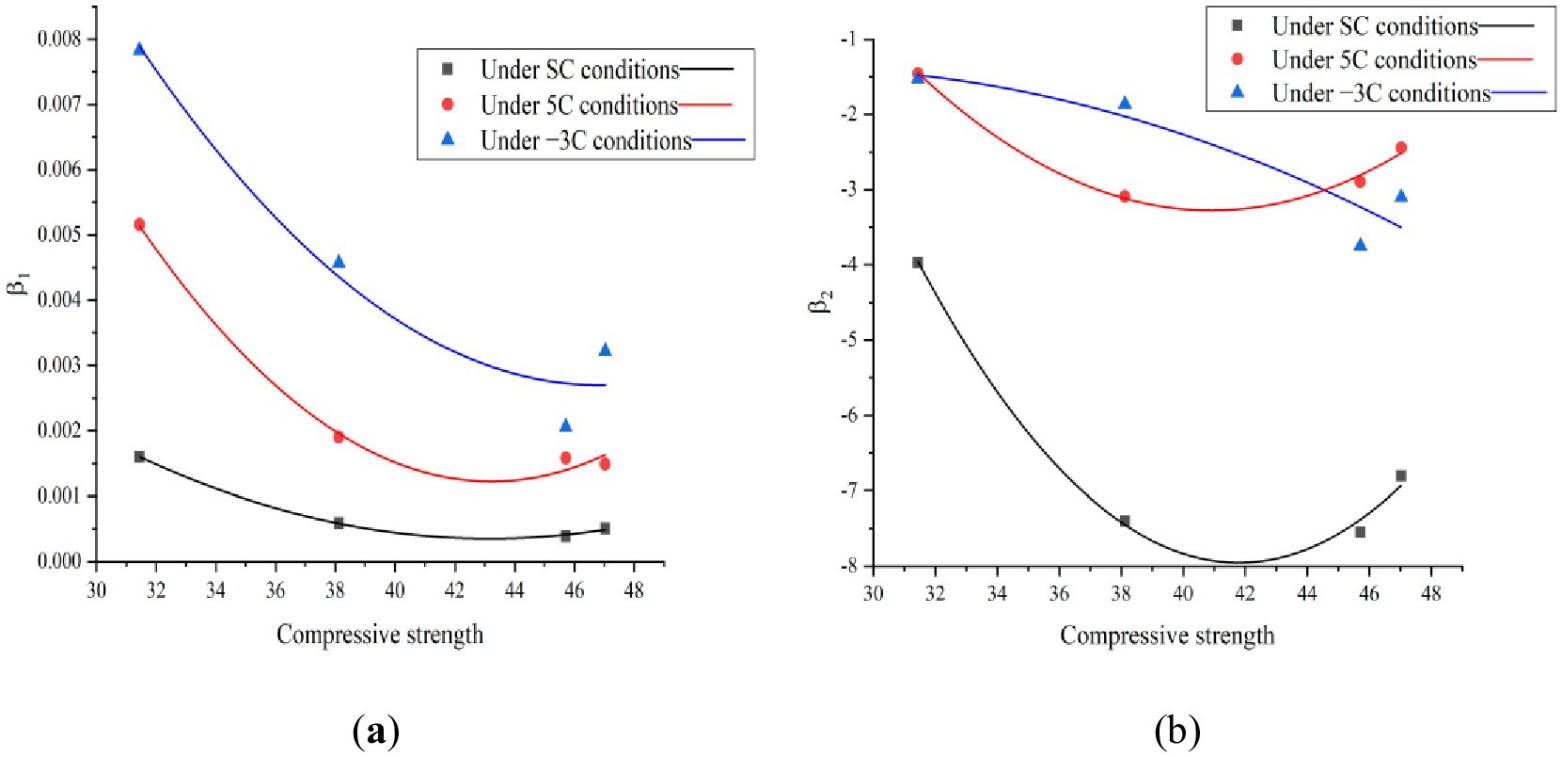
Relationship between *β*_1_ and *β*_2_ and compressive strength.

In the F–T test, the standard curing, 5 °C curing, and −3 °C curing conditions for 28, 56, and 84-d, respectively, are selected to compare the FRC. To ensure the same CSC, the relationship between the material parameters and compressive strength in the FTDM of concrete was studied. According to Section 3.1, the Pei model can predict the evolution law of concrete FTD more accurately. Therefore, in this study, the material parameters (*β*_1_ and *β*_2_) in the Pei model are fitted with respect to the CSC under standard curing conditions for 28-d. The FTDM of concrete was established considering the 28-d compressive strength under standard curing conditions. The fitted curves of the material parameters (*β*_1_ and *β*_2_) and CSC are shown in Fig 14. The fitting equations and correlation coefficients are listed in Table 8. From Fig 14, it can be seen that *β*_1_ and *β*_2_ show a trend of decreasing and then increasing with the increase of CSC. This is opposite to the trend of increase and then decrease of FRC with the increase of air content. It can be seen that and are related to the FRC, and the better the FRC, the lower the values of *β*_1_ and *β*_2_ are. In addition, it can be seen from Fig 14(b) that the fitted curves for −3 °C curing conditions show a slowly decreasing trend, which was different from that of the standard curing and 5 °C curing conditions, and this was related to that of the fitted curves in Table 7.

**Table 8:** Fitting equation of material parameters *β*_1_ and *β*_2_ and compressive strength in Pei model.

Curing condition	Group	Relational expression	*R* ^2^
standard curing	A-1–A-4	$\beta_{1}=9.098\times 10^{-6}{f_{c}}^{2}-7.852\times 10^{-4}f_{c}+0.017$	0.998
		$\beta_{2}=0.037{f_{c}}^{2}-3.118f_{c}+57.199$	0.996
5 °C curing	A-1–A-4	$\beta_{1}=2.823\times 10^{-5}{f_{c}}^{2}-0.002f_{c}+0.054$	0.986
		$\beta_{2}=0.204{f_{c}}^{2}-1.668f_{c}+30.842$	0.996
−3 °C curing	A-1–A-4	$\beta_{1}=2.200\times 10^{-5}{f_{c}}^{2}-0.002f_{c}+0.051$	0.960
		$\beta_{2}=-0.005{f_{c}}^{2}-0.290f_{c}-5.312$	0.860

Table 8 indicates that the correlation coefficients of the fitted curves all exceed 0.85, implying a good fit. Four groups of concrete specimens under standard curing conditions are considered as examples. The FTDM of concrete is given by Eq (9):

$$\begin{array}{cc} D & =1-[1-(0.037{f_{c}}^{2}-3.118f_{c}+57.199)\times N\\ & \times (1-9.098\times 10^{-6}{f_{c}}^{2}+7.852\times 10^{-4}f_{c}-0.017){]}^{\frac{1}{1-9.098\times 10^{-6}{f_{c}}^{2}+7.852\times 10^{-4}f_{c}-0.017}} \end{array}$$ (9)

## 5. Conclusions

In this study, the changes in the mechanical properties and FRC under 5 °C curing and −3 °C curing conditions are investigated. This study provides a theoretical framework and actionable guidance for constructing concrete under consistently low and below-freezing curing conditions. The main conclusions drawn are as follows.

1. The CSCs of group A-2 under the standard curing, 5 °C curing, and −3 °C curing conditions for 28, 56, and 84-d are 45.7, 46, and 44.8 MPa, respectively. Under the 5 °C curing and −3 °C curing conditions, longer curing times are required to reach the 28-d CSC obtained under the standard curing condition. Moreover, a distinct age lag phenomenon is observed. It becomes clear that extending the curing period allows concrete subjected to low and sub-zero temperatures to achieve compressive strength comparable to that of concrete cured under standard conditions. Under the same curing conditions, the CSC decreases with increasing air content.
2. In this study, the FRC under the standard curing, 5 °C curing, and −3 °C curing conditions for 28, 56, and 84-d is compared, respectively. The FRC under the 5 °C curing and −3 °C curing conditions is considerably weaker than the FRC under standard curing conditions. The FTCs of the four concrete groups, A-1–A-4, cured under standard conditions and satisfying the failure criteria are 250, 300, 200, and 125, respectively. As the air content increases, the FRC first increases and then decreases. Too little or too much AEA was not conducive to the frost RFC. When the air content was 3.6%, concrete can withstand the maximum number of FTCs when the failure criteria are satisfied (i.e., the best frost resistance).
3. Under the three curing conditions, the T_2_ spectral curve of concrete gradually moves to the right as the number of FTCs increases. When the air content is constant, the porosity of the concrete increases with decreasing curing temperature. The porosities of concrete group A-2 under the 5 °C curing and −3 °C curing conditions without F–T are 1.63 and 2.17 times greater than the porosity of this group cured under standard conditions, respectively. The primary cause lies in the FTC process, where the presence of saturated water within the concrete’s pores leads to the generation of freezing expansion forces. These forces occur within the fatigue-inducing environment of FTC and subsequently contribute to an amplified quantity of enlarged pores within the concrete. As the number of FTCs increases, the proportions of harmless and less harmful pores decrease, whereas those of the harmful and harmful pores increase.
4. Based on the FTDMs proposed in previous studies, an FTDM of concrete considering the 28-d CSC under standard curing conditions is developed under different curing conditions. The proposed model can predict the law of FTD and the deterioration of concrete under the 5 °C curing and −3 °C curing conditions according to the 28-d CSC under standard curing conditions. This study is anticipated to be used as reference for determining the FRC cured under different temperatures.

## References

[1] Qiang G, Fu W, Yi G. Study on the performance of an ultra-low energy building in the Qinghai-Tibet Plateau of China. J Build Eng, 2023, 70,106345. doi: 10.1016/j.jobe.2023.106345

[2] Zhao L, Zou D, Hu G. Changing climate and the permafrost environment on the Qinghai–Tibet (Xizang) plateau. Permafrost Periglac. 2020,31, 396–405. doi: 10.1002/ppp.2056

[3] Saludung A, Azeyanagi T, Ogawa Y. Mechanical and microstructural evolutions of fly ash/slag-based geopolymer at high temperatures: Effect of curing conditions. Ceram Int, 2023, 49(2), 2091–2101. doi: 10.1016/j.ceramint.2022.09.175

[4] Zajac M, Hilbig H, Bullerjahn F. Reactions involved in carbonation hardening of Portland cement: effect of curing temperature. J Sustain Cem-Based, 2023, 12(9),1107–1125. doi: 10.1080/21650373.2022.2163432

[5] Akçaoğlu T, Cubukcuoglu B, Hussain R. Effects of matrix quality and curing conditions on microcracking behaviour of modified concrete. Eur J Environ Civ En, 2023, 27(5), 2248–2260. doi: 10.1080/19648189.2022.2119604

[6] Landers K, Streletskiy D. (Un) frozen foundations: A study of permafrost construction practices in Russia, Alaska, and Canada. Ambio, 2023, 52, 1170–1183. doi: 10.1007/s13280-023-01866-9 37115428 PMC10247633

[7] Li R, Zhang M, Konstantinov P, Pei W. Tregubov O, Li G. Permafrost degradation induced thaw settlement susceptibility research and potential risk analysis in the Qinghai-Tibet Plateau. Catena 2022, 214,106239. doi: 10.1016/j.catena.2022.106239

[8] Streletskiy D. A. Clemens S, Lanckman J. P. The costs of Arctic infrastructure damages due to permafrost degradation. Environ Res Lett, 2023, 18, 015006. doi: 10.1088/1748-9326/acab18

[9] Manawadu A, Qiao P, Wen H, Freeze-thaw durability of shotcrete-concrete interface bonds in tension. Cold Reg Sci Technol, 2023, 103798. doi: 10.1016/j.coldregions.2023.103798

[10] Yi Y, Zhu D, Guo S, Zhang Z. A review on the deterioration and approaches to enhance the durability of concrete in the marine environment. Cement Concrete Comp. 2020, 113,103695. doi: 10.1016/j.cemconcomp.2020.103695

[11] Mansour M. A, Ismail M. H. B, Imran Latif Q. B. A systematic review of the concrete durability incorporating recycled glass. Sustainability, 2023, 15(4), 3568. doi: 10.3390/su15043568

[12] Beskopylny A. N, Shcherban E. M, Stel’makh S. A. Influence of Variatropy on the Evaluation of Strength Properties and Structure Formation of Concrete under Freeze-Thaw Cycles. J Compos Sci, 2023, 7(2), 58. doi: 10.3390/jcs7020058

[13] Rashidi Y, Korayem A. H, Farsi S. Utilizing halloysite nanotube to enhance the properties of cement mortar subjected to freeze-thaw cycles. J Build Eng, 2023, 75,106832. doi: 10.1016/j.jobe.2023.106832

[14] Tanyildizi H, Durability of concrete exposed to combined freeze-thaw, sulfate, and acid attacks after two years. Revista de la construccion, 2023, 22(1),102–121. doi: 10.7764/rdlc.22.1.102

[15] Raczkiewicz W, Bacharz M, Bacharz K. Reinforcement Corrosion Testing in Concrete and Fiber Reinforced Concrete Specimens Exposed to Aggressive External Factors. Materials, 2023, 16(3): 1174. doi: 10.3390/ma16031174 36770179 PMC9919189

[16] Adu-Amankwah S, Haha M.B, Zajac M, Black L, Skocek J. Relationship between cement composition and the freeze–thaw resistance of concretes, Adv. Cem. Res. 2018, 30, 387–397. doi: 10.1680/jadcr.17.00138

[17] Islam M.M, Tarequl Alam M, Islam M.S. Effect of fly ash on freeze–thaw durability of concrete in marine environment, Aust. J. Struct. Eng. 2018, 19, 146–161. doi: 10.1080/13287982.2018.1453332

[18] Wawrzenczyk J, Molendowska A, Juszczak T. Determining k-value with regard to freeze-thaw resistance of concretes containing GGBS, Materials, 2018, 11(12), 2349. doi: 10.3390/ma11122349 30469513 PMC6316923

[19] Correia V, Gomes Ferreira J, Tang L, Lindvall A. Luping Tang, Anders Lindvall. Effect of the addition of GGBS on the frost scaling and chloride migration resistance of concrete, Appl. Sci, 2020, 10(11), 3940. doi: 10.3390/app10113940

[20] Alsaif A, Bernal S.A, Guadagnini M, Pilakoutas K. Freeze-thaw resistance of steel fibre reinforced rubberised concrete, Constr. Build. Mater, 2019, 195, 450–458. doi: 10.1016/j.conbuildmat.2018.11.103

[21] Berto L, Saetta A, Talledo D. A New Bond Degradation Model for Freeze–Thaw-Damaged Reinforced Concrete. J Struct Eng, 2023, 149(8),04023109. doi: 10.1061/jsendh.steng-11577

[22] Bai J, Zhao Y, Shi J, He X. Damage degradation model of aeolian sand concrete under freeze–thaw cycles based on macro-microscopic perspective. Constr Build Mater, 2022, 327,126885. doi: 10.1016/j.conbuildmat.2022.126885

[23] Pei W. Model analysis of freeze-thaw damage deterioration of concrete based on fatigue damage theory. Railw Constr Technol, 2014, 5,51–54. (in Chinese)

[24] Manawadu A, Qiao P, Wen H. Freeze-thaw durability of shotcrete-concrete interface bonds in tension. Cold Reg Sci Technol, 2023,103798. doi: 10.1016/j.coldregions.2023.103798

[25] Zaghian S, Martín‐Pérez B, Almansour H. Nonlinear finite element modeling of bridge piers under the combined effect of corrosion, freeze–thaw cycles, and service load. Struct Concrete, 2023,1464–4177. doi: 10.1002/suco.202200370

[26] Du L, Folliard K. Mechanisms of air entrainment in concrete. Cement Concr Res, 2005, 35,1463–1471. doi: 10.1016/j.cemconres.2004.07.026

[27] Shah H, Yuan Q, Zuo S. Air entrainment in fresh concrete and its effects on hardened concrete-a review. Constr Build Mater, 2021, 274,121835. doi: 10.1016/j.conbuildmat.2020.121835

[28] Barfield M, Ghafoori N. Air-entrained self-consolidating concrete: A study of admixture sources. Constr Build Mater. 2012, 26,490–496. doi: 10.1016/j.conbuildmat.2011.06.049

[29] Şahin Y, Akkaya Y, Boylu F, Taşdemir MA. Characterization of air entraining admixtures in concrete using surface tension measurements. Cement Concrete Comp, 2017, 82,95–104. doi: 10.1016/j.cemconcomp.2017.03.023

[30] Hasholt MT. Air void structure and frost resistance: a challenge to Powers’ spacing factor. Mater Struct. 2014, 47,911–923. doi: 10.1617/s11527-013-0102-9

[31] Blikharskyy Z, Markiv T, Sobol K. The effect of air-entraining agent on the properties of mortars. Arch Civ Eng, 2023,147–156-147–156. doi: 10.24425/ace.2023.146072

[32] Kia A. Freeze-thaw durability of air-entrained high-strength clogging resistant permeable pavements. Constr Build Mater, 2023, 400,132767. doi: 10.1016/j.conbuildmat.2023.132767

[33] Anwar M, Emarah D. A. Chloride permeability through different specimen surfaces of blast-furnace slag cement concrete with and without air-entraining agent. Appl Eng Sci, 2023, 15,100134. doi: 10.1016/j.apples.2023.100134

[34] Stergiou K, Ntakolia C, Varytis P, Koumoulos E, Karlsson P, Moustakidis S. Enhancing property prediction and process optimization in building materials through machine learning: A review, Constr. Build. Mater, 2023, 220, 112031. doi: 10.1016/j.commatsci.2023.112031

[35] GB 175-2007.General purpose Portland Cement. Standards Press of China.2019

[36] GB/T14684-2022. Pebbles and gravel for Construction. China Building Industry Press.2022

[37] GB/T14684-2022. Construction sand. China Building Industry Press.2022

[38] JGJ 55–2011. Specification for mix proportion design of ordinary concrete. China Architecture and Building Press. 2011.

[39] Li J, Kaunda RB, Zhou K. Experimental investigations on the effects of ambient freeze-thaw cycling on dynamic properties and rock pore structure deterioration of sandstone. Cold Reg Sci Technol, 2018, 154,133–141. doi: 10.1016/j.coldregions.2018.06.015

[40] Zhang J, Deng H, Deng J, Gao R. Fractal Analysis of Pore Structure Development of Sandstone: A Nuclear Magnetic Resonance Investigation. IEEE Access, 2019, 7,47282–47293. doi: 10.1109/ACCESS.2019.2909782

[41] Dai J, Wang Q, Zhang B. Frost resistance and life prediction of equal strength concrete under negative temperature curing. Constr Build Mater, 2023, 396, 132278. doi: 10.1016/j.conbuildmat.2023.132278

[42] Jin W, Jiang L, Han L. Influence of curing temperature on freeze-thaw resistance of limestone powder hydraulic concrete. Case Stud Constr Mat, 2022,17,2214–5095. doi: 10.1016/j.cscm.2022.e01322

[43] Chen J, Li Y, Effects of curing conditions with different temperature and humidity on damage evolution of concrete during freeze–thaw cycling. Mater Struct, 2022, 55, 80. doi: 10.1617/s11527-022-01921-z

[44] Zhang K, Zhou J, Yin Z, Experimental study on mechanical properties and pore structure deterioration of concrete under freeze–thaw cycles. Materials, 2021, 14(21), 6568. doi: 10.3390/ma14216568 34772090 PMC8585203

[45] Luo Q, Liu D, Qiao P. Microstructural damage characterization of concrete under freeze-thaw action. Int J Damage Mech, 2018, 27(10), 1551–1568. doi: 10.1177/1056789517736573

[46] Wu Z, Lian H. High performance concrete, China Railway Publishing House, 1999

[47] Bu J, Xu H, Chen X. Freeze-thaw damage of ultra fine dredged sand concrete based on microstructure characteristics. Case Stud Constr Mat, 2024, e03666, doi: 10.1016/j.cscm.2024.e03666

[48] Ma D, Zhang W, Wang X. Effects of curing temperature on mechanical properties and pore size distribution of cement clay modified by metakaolin and basalt fiber. J Build Eng, 2023, 68, 106232. doi: 10.1016/j.jobe.2023.106232

